# Total synthesis and biological evaluation of fluorinated cryptophycins

**DOI:** 10.3762/bjoc.8.231

**Published:** 2012-11-23

**Authors:** Christine Weiß, Tobias Bogner, Benedikt Sammet, Norbert Sewald

**Affiliations:** 1 Organic and Bioorganic Chemistry, Department of Chemistry, Bielefeld University, PO Box 100131, 33501 Bielefeld, Germany

**Keywords:** antimitotic drug, cytotoxicity, depsipeptide, fluorinated natural product analogues, structure-activity relationship

## Abstract

Cryptophycins are cytotoxic natural products that exhibit considerable activities even against multi-drug-resistant tumor cell lines. As fluorinated pharmaceuticals have become more and more important during the past decades, fluorine-functionalized cryptophycins were synthesized and evaluated in cell-based cytotoxicity assays. The unit A trifluoromethyl-modified cryptophycin proved to be highly active against KB-3-1 cells and exhibited an IC_50_ value in the low picomolar range. However, the replacement of the 3-chloro-4-methoxyphenyl-substituent in unit B by a pentafluorophenyl moiety resulted in a significant loss of activity.

## Introduction

**Figure 1:**
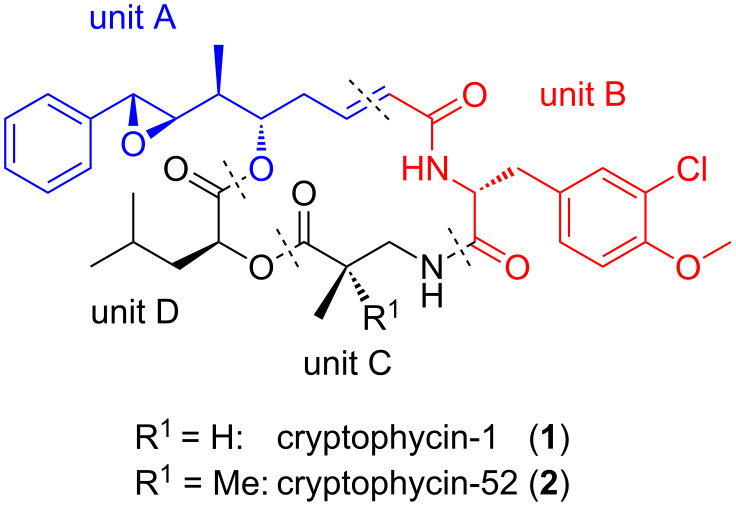
Structures of cryptophycin-1 (**1**) and -52 (**2**).

Cryptophycins form a class of cytotoxic sixteen-membered macrocyclic depsipeptides. Cryptophycin-1 (**1**) was isolated for the first time in 1990 from cyanobacteria *Nostoc* sp. ATCC 53789 [1] (Figure 1). Moore et al. isolated cryptophycin-1 from the related *Nostoc* strain GSV 224, investigated the stereochemistry, and described the cytotoxicity [2]. At the same time Kobayashi et al. succeeded in a full structural analysis and described the first total synthesis of another member of the cryptophycin family [3–4]. Twenty-eight naturally occurring cryptophycins have been isolated up to this day [5–7], while numerous synthetic analogues have been synthesized in the frame of structure–activity-relationship studies [8–9]. Cryptophycins display remarkable biological activity against multi-drug-resistant (MDR) tumor cells. Such tumor cells express a P-glycoprotein, a drug efflux pump that transports xenobiotics out of the cell. A synthetic analogue, cryptohycin-52 (**2**, LY355703), has been investigated in clinical trials. However, this development was discontinued because of neurotoxic side effects and lacking efficacy in vivo [10–11].

Fluorinated drugs are gaining increasing importance, and currently about 20% of all pharmaceuticals on the world market contain fluorine substituents [12–13]. Fluorination is supposed to enhance bioavailability and receptor selectivity. The van der Waals-radius of a fluorine substituent (1.47 Å) lies between the value of a hydrogen substituent (1.20 Å) and an oxygen substituent (1.52 Å). However, despite this similarity in size, a fluorine substituent exerts considerable electronic effects due to the high electronegativity. A trifluoromethyl substituted analogue of epothilone, another important tubulin-binding cytotoxic drug, was shown to retain the cytotoxic activity of the parent compound. At the same time nonspecific side effects due to oxidative degradation were prevented by the introduction of the CF_3_ group [14–15]. Likewise, partially fluorinated taxoids, analogues of paclitaxel and docetaxel, displayed biological activity even exceeding that of the parent nonfluorinated compounds [16]. The interesting biological profile of fluorinated cytotoxic agents prompted us to synthesize partially fluorinated analogues of cryptophycins.

The depsipeptidic character of the cryptophycins suggests four different fragments to be assembled in the total synthesis, named unit A–D (Figure 1). Unit A is an α,β-unsaturated δ-hydroxy acid that usually also contains a benzylic epoxide or a benzylic double bond. Unit B represents a chlorinated *O*-methyl-D-tyrosine derivative, while unit C is a β^2^-amino acid, usually β^2^-homoalanine. Finally, unit D is leucic acid, the hydroxy analogue of leucine. Numerous synthetic analogues have been obtained in the frame of structure–activity-relationship studies (SAR-studies), as reviewed in [17–18].

Unit A *para*-alkoxymethyl derivatives of cryptophycin-52 have been synthesized and were shown to retain cytotoxicity even against MDR tumor cell lines [19]. The introduction of a fluorine substituent in the same position also provides a cytotoxic analogue, albeit with decreased biological activity by a factor of 5 [8].

**Figure 2:**
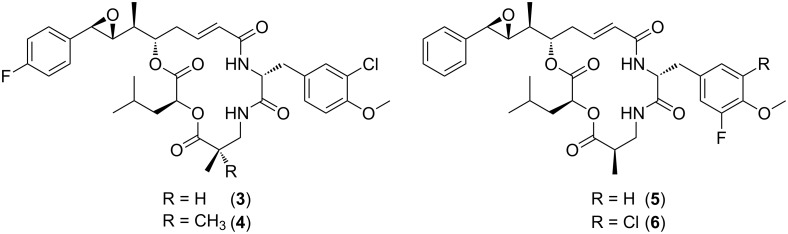
Fluorinated derivatives of cryptophycin-1 and -52 [20–22].

In unit B the chlorine and the methoxy substituents at the D-tyrosine residue were crucial for high antimitotic activity [17–18]. Moore et al. patented the synthesis of fluorinated analogues of cryptophycin-1 and cryptophycin-52 [20]. In particular, derivative **3** was shown to retain biological activity (IC_50_ = 39 pM) and was active against the tumor cell line KB-3-1 [21] (Figure 2). The chlorohydrin derived from **4** that also contained a fluorine substituent in the *para*-position of the unit A phenyl ring was patented as a promising candidate [22].

In the frame of our on-going SAR studies on cryptophycins [19,23–30], we envisaged the synthesis of analogues of cryptophycin-52 with a *para*-trifluoromethyl substituent at the unit A aryl ring. In addition, we targeted the replacement of the unit B by a D-pentafluorophenylalanine residue.

## Results and Discussion

### Cryptophycin-52 with a *para*-trifluoromethyl substituted unit A

**Scheme 1:**
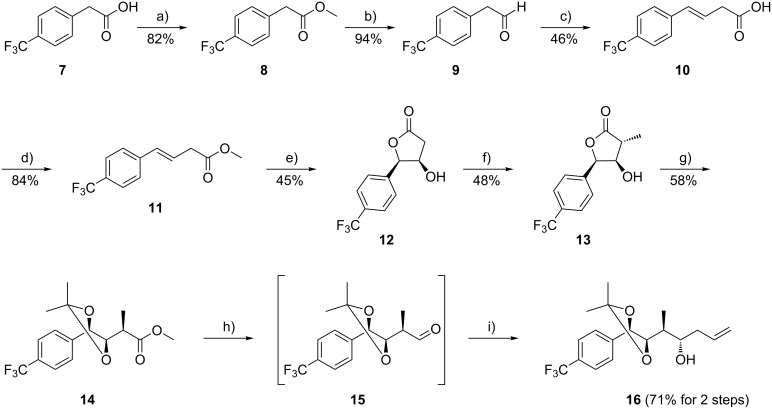
Access to the trifluoromethyl substituted unit A-building block **16**. Reagents and conditions: (a) SOCl_2_, MeOH, 0 °C → rt, 16 h; (b) DIBAL-H, CH_2_Cl_2_, −78 °C, 3.5 h; (c) malonic acid, piperidine, AcOH, DMSO, 65 °C, 1.5 h; (d) SOCl_2_, MeOH, 0 °C → rt, 1 h; (e) K_2_CO_3_, K_2_OsO_4_∙2H_2_O, K_3_[Fe(CN)_6_], (DHQD)_2_-PHAL, CH_3_SO_2_NH_2_, *t*-BuOH/H_2_O, 0 °C, 42 h; (f) LDA, MeI, THF, −78 °C, 3 d; (g) (CH_3_)_2_C(OCH_3_)_2_, MeOH, Amberlyst-15^®^, rt, 8 d; (h) DIBAL-H, CH_2_Cl_2_, −78 °C, 4.5 h; (i) AllylSnBu_3_, MgBr_2_∙Et_2_O, CH_2_Cl_2_, −78 °C, 15 h.

The synthesis of the *para*-trifluoromethyl substituted unit A started with a modified Knoevenagel condensation [23,31]. The required aldehyde **9** was obtained by DIBAL-H reduction of the corresponding methyl ester **8** and was found to decompose upon chromatographic purification (Scheme 1). However, it can usually be employed in the Knoevenagel condensation without purification. Reaction of **9** with malonic acid in the presence of piperidine/acetic acid gave the β,γ-unsaturated carboxylic acid **10**. The latter compound was transformed into the methyl ester by treatment with SOCl_2_ in methanol. The resulting ester **11** could then be directly employed without purification in the asymmetric dihydroxylation with osmium tetroxide and (DHQD)_2_PHAL, in close analogy to a previously published procedure [23]. The initially formed vicinal diol cyclizes under the reaction conditions to give lactone **12** in enantiomerically pure form (chiral HPLC: Chiralpak OD^®^). Deprotonation of **12** with 2.5 equiv of LDA, followed by treatment with iodomethane furnished the α-methyl substituted lactone **13**. Treatment of this compound with acetone dimethyl acetal in methanol in the presence of an acidic ion exchanger resulted in acetonide protection of the vicinal diol, accompanied by methyl ester formation. The methyl ester **14** was subsequently reduced with DIBAL-H to give the aldehyde **15**. In order to avoid epimerisation, this aldehyde was not purified, but filtered through Celite only and then reacted with allyl-tri-*n*-butyltin to give the homoallyl alcohol **16**. The magnesium bromide diethyl etherate mediated allylation proceeded under substrate control and with complete diastereoselectivity [23,32].

**Scheme 2:**
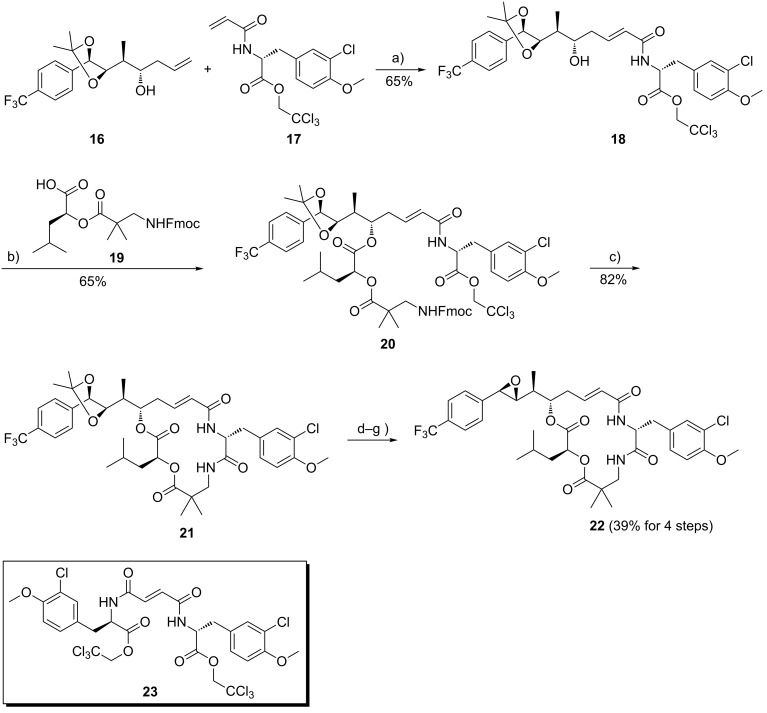
Assembly of units A–D and macrocyclization, followed by diol-epoxide transformation to give the trifluoromethyl substituted analogue **22** of cryptophycin-52. Reagents and conditions: (a) Grubbs II catalyst, CH_2_Cl_2_, reflux, 16 h; (b) **19**, DMAP, NEt_3_, 2,4,6-trichlorobenzoylchloride, THF, 0 °C, 1 h; (c) piperidine, DMF, rt, 16 h; (d) TFA, CH_2_Cl_2_, H_2_O, 0 °C, 3 h; (e) (CH_3_O)_3_CH, PPTS, CH_2_Cl_2_, rt, 2 h; (f) AcBr, CH_2_Cl_2_, rt, 4 h; (g) K_2_CO_3_, DME, ethylene glycol, rt, 3 min.

Cross-metathesis of homoallyl alcohol **16** with the unit B derived acrylamide **17** provided the α,β-unsaturated δ-hydroxy carboxamide **18** (Scheme 2). In order to bring about complete metathesis of **16**, the acrylamide **17** had to be employed in 1.2-fold excess, which resulted in a contamination of the cross-metathesis product **18** with minor amounts of the homo-coupling product **23**. The latter could not be separated by flash chromatography on this stage, but did not interfere with the subsequent Yamaguchi esterification of **18** with the unit C–D segment **19** and was removed on this stage [33]. Fmoc cleavage of the *seco*-depsipeptide **20** liberated the free amino group of unit C, which under the reaction conditions displaced the trichloroethylester of unit B resulting in macrocyclization according to Moher et al. [34]. In the final steps the dioxolane ring of **21** was cleaved with trifluoroacetic acid in the presence of water. The resulting vicinal diol was not purified, but reacted with a large excess of trimethyl orthoformate. The cyclic orthoester resulting from this transformation was directly subjected to reaction with acetyl bromide to form a bromohydrin formate. This was then treated with a potassium carbonate/ethylene glycol/dimethoxyethane-emulsion to bring about cleavage of the formyl ester accompanied by epoxide formation as previously described by us [19]. The trifluoromethyl substituted cryptohycin-52 analogue **22** was obtained in a yield of 39% over the final four steps. It was purified by column chromatography, followed by lyophilization.

### Cryptophycin-52 with D-pentafluorophenylalanine as unit B

**Scheme 3:**
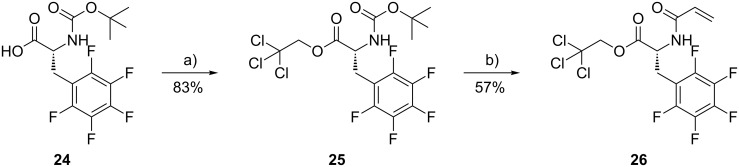
Synthesis of the pentafluorophenylalanine building block **26**. Reagents and conditions: (a) pyridine, trichloroethanol, DCC, CH_2_Cl_2_, 0 °C, 20 h; (b) 1. TFA, rt, 2 h; 2. NEt_3_, acryloylchloride, CH_2_Cl_2_, 0 °C, 7 h.

The *N*-acryloyl derivative **26** of D-pentafluorophenylalanine was obtained by carbodiimide esterification of commercially available Boc-D-pentafluorophenylalanine (**24**) with trichloroethanol, followed by cleavage of Boc and reaction with acryloylchloride in the presence of base [19] (Scheme 3).

**Scheme 4:**
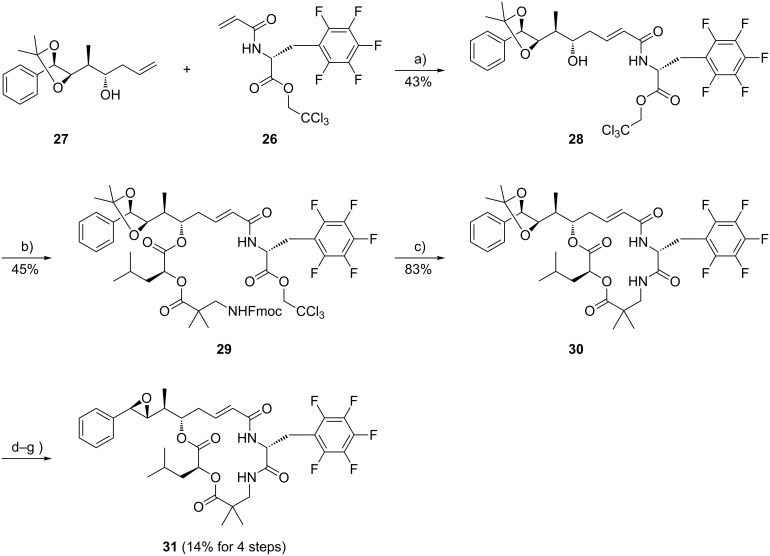
Convergent synthesis of the pentafluorinated cryptophycin **31**. Reagents and conditions: (a) Grubbs II catalyst, CH_2_Cl_2_, reflux, 16 h; (b) **19**, DMAP, NEt_3_, 2,4,6-trichlorobenzoylchloride, THF, 0 °C, 1 h; (c) piperidine, DMF, rt, 16 h; (d) TFA, CH_2_Cl_2_, H_2_O, 0 °C, 3 h; (e) (CH_3_O)_3_CH, PPTS, CH_2_Cl_2_, rt, 2 h; (f) AcBr, CH_2_Cl_2_, rt, 4 h; (g) K_2_CO_3_, DME, ethylene glycol, rt, 3 min.

The cryptophycin analogue with D-pentafluorophenylalanine as unit B was synthesized by the same convergent route as described for derivative **22**. Homoallyl alcohol **27** [23] was reacted with the D-pentafluorophenylalanine derivative **26** in a cross-metathesis reaction in the presence of Grubbs II catalyst (Scheme 4). The resulting α,β-unsaturated δ-hydroxy carboxamide **28**, representing units A and B was then esterified with **19** under Yamaguchi conditions with 2,4,6-trichlorobenzoylchloride and triethylamine in the presence of catalytic amounts of DMAP. Macrocyclization was brought about by cleavage of the Fmoc protecting group from the unit C amino group, which concomitantly displaced the trichloroethylester at unit B to result in the macrocyclic product **30** [34]. Cleavage of the dioxolane liberated the vicinal diol, which was then subjected to the final diol-epoxide transformation to provide the cryptophycin-52 analogue **31** in a yield of 14% over the final four steps.

The biological activities of the fluorine-functionalized cryptophycin analogues were determined in a resazurin assay with the tumor cell line KB-3-1 and its MDR correlate KB-V1. The IC_50_ values of the fluorinated cryptophycins **22** and **31** were compared to cryptophycin-52 in Table 1 [17]. While the cytotoxicity of the unit A-modified analogue **22** against the tumor cell line KB-3-1 was only by about a factor of 4 decreased compared to cryptophycin-52, the pentafluorophenylalanine-containing derivative **31** was much less active. A significant loss in activity of both analogues against the MDR cell line KB-V1 was observed. The degree of activity against MDR tumor cells can be described by the resistance factor *F*_R_, which is defined as the ratio of the IC_50_ value for the MDR cell line and the value for the nonresistant cell line. A high *F*_R_ means a high loss of activity due to the cellular resistance mechanisms. Analogue **22** exhibited a high *F*_R_ value whereas compound **31** showed a lower loss of activity.

**Table 1:** Cytotoxicity of the fluorinated cryptophycins **22** and **31** in comparison to cryptophycin-52 (**2**).

	IC_50_ [pM] (KB-3-1)	IC_50_ [nM] (KB-V1)	*F*_R_

**2**	15.5	0.26	16.7
**22**	66.0	10.1	153
**31**	2970	98.4	33

## Conclusion

The synthesis of selectively fluorinated cryptophycin-52 analogues succeeded and both target compounds could be obtained. The two analogues were less active, both against the tumor cell line KB-3-1 and its MDR subclone KB-V1. This fact was quite surprising because the fluorinated cryptophycins were expected to display higher lipophilicity compared to the parent compound cryptophycin-52 and, therefore, exhibit equal or even higher activities. In contrast, more amphiphilic or polar compounds are usually good substrates for the P-glycoprotein efflux pump resulting in a decreased bioactivity.

## Supporting Information

File 1 Full experimental procedures and detailed analytical data for the synthesis of all compounds.
