# Supplementary material for: Total synthesis and biological evaluation of fluorinated cryptophycins
Source: Beilstein J Org Chem. 2012 Nov 23;8:2060–6. doi: 10.3762/bjoc.8.231 (PMC3511040; doi:10.3762/bjoc.8.231)
Supplement: File 1 — Full experimental procedures and detailed analytical data for the synthesis of all compounds. [file Beilstein_J_Org_Chem-08-2060-s001.pdf]

Supporting Information

for

Total synthesis and biological evaluation of fluorinated cryptophycins

Christine Weiß, Tobias Bogner, Benedikt Sammet and Norbert Sewald*

Address: Organic and Bioorganic Chemistry, Department of Chemistry, Bielefeld University, PO Box 100131, 33501 Bielefeld, Germany

Email: Norbert Sewald* - norbert.sewald@uni-bielefeld.de

* Corresponding author

Full experimental procedures and detailed analytical data for the synthesis of all compounds

Table of contents

1 General information **2**

2 General procedures **4**

General procedure GP1 – Condensation of units A-B with units C–D 4

General procedure GP2 – Macrolactamization 5

General procedure GP3 – Cleavage of the acetonide 5

General procedure GP4 – Synthesis of the cyclic orthoformate 5

General procedure GP5 – Synthesis of the bromohydrin formate 6

General procedure GP6 – Synthesis of the epoxide 6

3 Experimental procedures and compound characterization **8**

3.1 Synthesis of the trifluoromethyl-substituted unit A building block 8

3.2 Synthesis of the pentafluorophenyl-substituted unit B building block 24

4 References **32**

1 General information

All chemicals were purchased from Sigma Aldrich (Hamburg, Germany), Acros (Geel, Belgium), Alfa Aesar (Ward Hill, USA) and VWR (Darmstadt, Germany), and were employed without additional purification. Moisture- and air-sensitive reaction steps were conducted in flame-dried glassware and under argon atmosphere. Dichloromethane and toluene were freshly distilled from CaH_2 and Na, respectively. Analytical RP-HPLC was performed on a Thermo Separation Products system equipped with a UV-6000 LP detector, a P-4000 pump, a Vydac high-performance guard column (C18) and a Phenomenex Jupiter 5 µm (C18; 250 × 4.6 mm). A flow rate of 0.7 mL min^{-1} using eluent A: H_2O/CH_3CN/TFA (95/5/0.1) and eluent B: CH_3CN/H_2O/TFA (95/5/0.1) was employed.

Method M1:

0 min	100% A	0% B
0–5 min	0% A	100% B
5–6 min	100% A	0% B
5–6.5 min	100% A	0% B

Preparative RP-HPLC was performed on a Thermo Separation Products system equipped with a UV-1000 detector, a P-4000 pump, a Vydac high-performance guard column (C18) and a Phenomenex Jupiter 10 µm 300 Å column (C18; 250 × 21.20 mm). A flow rate of 7.5 mL min^{-1} using eluent A: H_2O/CH_3CN/TFA (95/5/0.1) and eluent B: CH_3CN/H_2O/TFA (95/5/0.1) was employed.

Method M2:

0–3 min	70% A	30% B
3–20 min	30% A	70% B
20–50 min	30% A	70% B
50–55 min	70% A	30% B

In addition, a Hitachi Merck LaChrom system equipped with a UV–vis L-7420 detector, a L-7150 pump, a Vydac high-performance guard column (C18) and a Phenomenex Jupiter 10 µm 300 Å column (C18; 250 × 21.20 mm) was used.

Method M3:

0–3 min	80% A	20% B
3–13 min	0% A	100% B
13–35 min	0% A	100% B
35–50 min	80% A	20% B

For the chiral analytical HPLC a Thermo Separation Products system equipped with a UV-6000 detector, a P-4000 pump and a Daicel Chiralpak 10 µm column (C18; 250 × 4.60 mm) was used. A flow rate of 1 mL min^{-1} using an eluent consisting of hexane/isopropanol (9/1) was employed (method M4).

MALDI-TOF mass spectra were measured on a Voyager DE Instrument (PE Biosystems, Weiterstadt, Germany) mounted with a 1.2 m flight tube. 2,5-Dihydroxybenzoic acid was used as the matrix. Depending on the mass range the ions were accelerated at 15 to 25 kV with the option of detecting positive or negative ions. The instrument default calibration was used for calibrating the mass axis. ESI experiments were performed on a Fourier Transform Ion Cyclotron Resonance (FT-ICR) mass spectrometer APEX III (Bruker Daltonik, Bremen, Germany) equipped

with a 7.0 T, 160 mm bore superconducting magnet (Bruker Analytik GmbH – Magnetics, Karlsruhe, Germany), infinity cell, and interfaced to an external (nano)ESI or MALDI ion source. Scan accumulation and Fourier transformation were done with XMASS NT (7.08) on a PC workstation, for further data processing DataAnalysis™ 3.4 was used. Optical rotation was measured on a DIP-360 digital polarimeter (Jasco, Groß-Umstadt, Germany) at 20–23 °C. NMR spectra were recorded at 298 K in $CDCl_3$ on a DRX 500 (^{1}H: 500 MHz, ^{13}C: 126 MHz, ^{19}F: 470 MHz), an Avance III 500 (^{1}H: 500 MHz, ^{13}C: 126 MHz, ^{19}F: 470 MHz) and an Avance 600 spectrometer (^{1}H: 600 MHz, ^{13}C: 151 MHz, ^{19}F: 564 MHz) (Bruker Biospin, Rheinstetten, Germany). The acronyms uA, uB, uC and uD describe signals pertaining to cryptophycin-units A–D.

2 General procedures

General procedure GP1 – Condensation of units A-B with units C–D

A mixture of the units A–B (1.0 equiv), the units C–D (1.0–1.5 equiv) and DMAP (0.25 equiv) was dried for several hours under high vacuum and then dissolved in absolute THF (10 mL/mmol) under an argon atmosphere. After addition of absolute triethylamine (2.0 equiv) the solution was cooled to 0 °C and then 2,4,6-trichlorobenzoylchloride (1.50–2.50 equiv) was added. The reaction mixture was stirred for one further hour at 0 °C, and the conversion was monitored by TLC. Then, 10% citric acid solution (17 mL/mmol) was added, the mixture was allowed to warm up to room temperature, and the aqueous phase was extracted three times with EtOAc (3 × 55 mL/mmol). The combined organic extracts were washed with saturated $NaHCO_3$ solution (25 mL/mmol) and brine (25 mL/mmol), dried over $MgSO_4$ and evaporated to dryness in vacuum. The crude product was purified by flash chromatography or preparative RP-HPLC.

General procedure GP2 – Macrolactamization

After addition of absolute piperidine (5.0 equiv) at room temperature to a solution of the acyclic depsipeptide (1.0 equiv in absolute DMF, 30 mL/mmol), the reaction mixture was stirred overnight in the dark, and the conversion was monitored by TLC. Subsequently, the reaction mixture was diluted with ethyl acetate (300 mL/mmol) and washed with H_2O (400 mL/mmol). The aqueous phase was extracted with ethyl acetate (3 × 200 mL/mmol) and the combined organic layers were washed with brine (100 mL/mmol), dried over $MgSO_4$ and evaporated to dryness in vacuum. The residue was purified by flash chromatography.

General procedure GP3 – Cleavage of the acetonide

Trifluoroacetic acid (10 mL/mmol) and water (5 drops) were added at 0 °C to a solution of the acetonide (1.0 equiv) in absolute dichloromethane (10 mL/mmol). The reaction mixture was stirred at this temperature while the reaction progress was monitored by TLC. After complete conversion (2–3 h) the solvent was removed in vacuum without heating the bath, the residue was dissolved in EtOAc (500 mL/mmol), and saturated $NaHCO_3$ solution (750 mL/mmol) was added. After phase separation the aqueous layer was extracted with EtOAc (3 × 250 mL/mmol). The combined organic layers were dried over $MgSO_4$, filtered and evaporated to dryness in vacuum. The resulting diol was dried under high vacuum and subjected to further reactions without purification.

General procedure GP4 – Synthesis of the cyclic orthoformate

The diol resulting from GP3 and PPTS (2.5 equiv) were dried overnight under high vacuum. Under argon atmosphere absolute dichloromethane (30 mL/mmol) and absolute trimethyl orthoformate (10 mL/mmol) were added. The reaction mixture was

stirred for two hours at room temperature while the reaction progress was monitored by TLC or analytical RP-HPLC. The reaction mixture was then filtered through a thin plug of silica gel, which was washed with EtOAc/CH_2Cl_2 (300 mL, 1:1 v/v). The combined filtrates were evaporated to dryness in vacuum. The resulting cyclic orthoformate was subjected to further conversions after drying under high vacuum without purification.

General procedure GP5 – Synthesis of the bromohydrin formate

The orthoformate (1.0 equiv) was dissolved in absolute CH_2Cl_2 (15 mL/mmol) and a 0.5 M solution of acetyl bromide in absolute CH_2Cl_2 (2.5 equiv acetyl bromide) was added. The resulting solution was stirred for four hours at room temperature while the reaction progress was monitored by TLC or analytical RP-HPLC. Subsequently, the reaction mixture was diluted with absolute CH_2Cl_2 (10 mL) and then poured into an ice-cold mixture of saturated $NaHCO_3$ solution and ice water (50 mL, 1:1, v/v). The mixture was washed with absolute CH_2Cl_2 and after phase separation the aqueous layer was extracted with CH_2Cl_2 (3 × 20 mL). The organic layers were combined, dried over $MgSO_4$, filtered and evaporated to dryness. The resulting bromohydrin formate was dried under high vacuum and subjected without further purification to the next reaction step.

General procedure GP6 – Synthesis of the epoxide

To the bromohydrin formate was added under vigorous stirring an emulsion of 0.2 M K_2CO_3 in ethylene glycol/DME. The reaction mixture was stirred for 3 min and then rapidly diluted with absolute CH_2Cl_2 (20 mL). The solution was transferred to a separating funnel containing ice-cold 0.5% aqueous $KHSO_4$ solution (20 mL). The reaction flask was rinsed with absolute CH_2Cl_2. The aqueous phase was extracted

with CH_2Cl_2 (2 × 20 mL) and the organic layers were combined and collected over solid $MgSO_4$. The organic layer should be separated from the aqueous phase as quickly as possible to avoid hydrolysis of the epoxide in aqueous acidic medium. For that reason, the formation of two clear phases was not awaited. After drying of the combined organic layers over $MgSO_4$ the solvent was evaporated in vacuum and the residue was purified by column chromatography.

3 Experimental procedures and compound characterization

3.1 Synthesis of the trifluoromethyl-substituted unit A building block

Methyl 4-trifluoromethylphenylacetate (8)

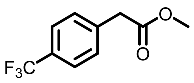

M = 218.17 g mol^{-1} $C_{10}H_9F_3O_2$

For the synthesis of **8** a procedure by Bodnar [1] was adapted. 4-Trifluoromethyl-phenylacetic acid (**7**) (5.00 g, 24.49 mmol, 1.0 equiv) was dissolved in MeOH p.a. (260 mL) and cooled to 0 °C. Thionylchloride (4.00 mL, 56.33 mmol, 2.3 equiv) was added during 30 min with cooling to 0 °C and the reaction mixture was stirred for further 30 min at the same temperature. The mixture was allowed to warm up to rt and was stirred overnight. Then, H_2O (50 mL) was added and stirring was continued for 10 min. Methanol was evaporated in vacuum (bath temperature = 30 °C) and the residue was taken up in H_2O (50 mL) and CH_2Cl_2 (200 mL). The phases were separated and the aqueous phase was extracted with CH_2Cl_2 (4 × 100 mL). The combined organic layers were washed with sat. $NaHCO_3$ solution (2 × 150 mL) and H_2O (100 mL) and subsequently dried over $MgSO_4$. The solvent was evaporated in vacuum and 4.37 g (82%) of the ester **8** was obtained as a colorless oil, which can be subjected to the next steps without further purification. The spectroscopic data were in agreement with literature values [1].

HPLC: t_R 4.380 min (method M1); ^{1}H NMR (500 MHz, $CDCl_3$, TMS) δ 3.69 (s, 2H, CH_2), 3.71 (s, 3H, CH_3), 7.40 (d, J = 8.1 Hz, 2H, $C^{ar}H$), 7.59 (d, J = 8.1 Hz, 2H, $C^{ar}H$).

(4-Trifluoromethylphenyl)acetaldehyde (9)

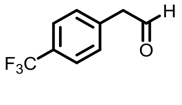

M = 188.15 g mol^{-1} $C_9H_7F_3O$

Adapting a procedure by Eißler et al. [2], methyl 4-trifluoromethylphenylacetate (**8**, 3.08 g, 14.11 mmol, 1.0 equiv) was dissolved in abs. CH_2Cl_2 (85 mL) and the mixture

was cooled to −78 °C. A solution of DIBAL-H (1.0 M in CH_2Cl_2, 42.0 mL, 42.00 mmol, 3.0 equiv) was added dropwise at −78 °C during 60 min and the solution was stirred at this temperature for 3.5 h. Then, it was cooled to −100 °C and the reaction was quenched by the addition of MeOH (18 mL). After warming up to rt, 1 M AcOH (160 mL) and hexane (160 mL) was added to the solution. It was vigorously stirred, the phases were separated, and the aqueous phase was extracted with petrol ether (3 × 160 mL). The combined organic phases were washed with 1 M AcOH (100 mL) and brine (100 mL), dried over $MgSO_4$ and filtered through Celite®. The solvents were evaporated in vacuum to dryness and 2.51 g (94%) of the product **9** was obtained as a clear colorless liquid that can be subjected to further reactions without purification.

HPLC: t_R 3.752 min (method M1); ^{1}H NMR (500 MHz, $CDCl_3$, TMS) δ 3.79 (d, J = 1.3 Hz, 2H, CH_2), 7.34 (d, J = 8.1 Hz, 2H, C^{ar}H), 7.63 (d, J = 8.1 Hz, 2H, C^{ar}H), 9.79 (t, J = 2.0 Hz, 1H, CHO); ^{13}C NMR (126 MHz, $CDCl_3$, TMS) δ 50.2 (CH_2), 124.0 (q, J = 272.5 Hz, CF_3), 125.8 (q, J = 3.8 Hz, $\underline{C}^{ar}HCCF_3$), 129.8 (q, J = 32.7 Hz, $\underline{C}^{ar}CF_3$), 130.0 (C^{ar}H), 135.9 (C^{ar}), 198.1 (CHO); ^{19}F NMR (470 MHz, $CDCl_3$) δ −62.7 (s, 3F, CF_3); IR (ATR) ṽ (cm^{-1}) 2832 w, 1723 s, 1618 m, 1418 m, 1320 s, 1160 s, 1108 s, 1065 s, 1018 s, 829 m, 732 w; HRMS–EI ($C_9H_7F_3O$): calcd 188.04490; found, 188.04420.

(*E*)-4-(*p*-Trifluoromethylphenyl)but-3-enoic acid (10)

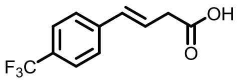
M = 230.18 g mol^{-1} $C_{11}H_9F_3O_2$

Adapting a procedure by Eißler et al. [2], malonic acid (1.05 g, 10.08 mmol, 2.2 equiv) was dissolved in DMSO (3.8 mL). Acetic acid (5 µL, 0.09 mmol, 0.2 equiv) in DMSO (110 µL), and piperidine (9 µL, 0.09 mmol, 0.2 equiv) in DMSO (110 µL) was added to the solution, which was heated to 65 °C and (4-trifluoromethylphenyl)-acetaldehyde (**9**, 0.86 g, 4.56 mmol, 1.0 equiv), dissolved in DMSO (2 mL), was added dropwise during 30 min. The reaction mixture was stirred for 1.5 h at 65 °C and then allowed to cool to rt. H_2O (20 mL) was added and the aqueous phase extracted with Et_2O (4 × 5 mL). The combined organic phases were washed with 5% $KHSO_4$ solution (5 mL, w/v) and brine (5 mL), and dried over $MgSO_4$, and the solvent was evaporated in vacuum. The residue was purified by column chromatography over silica gel (petrol ether/EtOAc = 8/1 + 1% AcOH). 481 mg (2.09 mmol, 46%) of the free acid was isolated as a yellowish solid after repeated co-evaporation with toluene. The spectroscopic data were in agreement with the literature [3].

R_f 0.34 (petrol ether/EtOAc = 5/1 + 1% AcOH); ^{1}H NMR (500 MHz, $CDCl_3$, TMS) δ 3.34 (dd, J = 7.1, 1.4 Hz, 2H, CH_2), 6.40 (dt, J = 15.9, 7.1 Hz, 1H, CH=C$\underline{H}$$CH_2$), 6.56 (dm, J = 15.9 Hz, 1H, C$\underline{H}$=CHCH_2), 7.47 (d, J = 8.2 Hz, 2H C^{ar}H), 7.57 (d, J = 8.2 Hz, 2H, C^{ar}H), 11.22 (br s, 1H, CO_2H).

Methyl (*E*)-4-[4-(trifluoromethyl)phenyl]but-3-enoate (11)

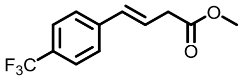
M = 244.21 g mol^{-1} $C_{12}H_{11}F_3O_2$

Adapting a procedure by Eißler et al. [2], (*E*)-4-(*p*-trifluoromethylphenyl)but-3-enoic acid (**10**, 0.48 g, 2.08 mmol, 1.0 equiv) was dissolved in MeOH (22 mL) and the solution was cooled to 0 °C. Thionyl chloride (227 µL, 3.12 mmol, 1.5 equiv) was

added dropwise. The reaction mixture was allowed to warm up to rt and stirred for 1 h at rt. It was then cooled to 0 °C and quenched with H_2O (16 mL). Stirring was continued for 10 min and then the aqueous phase was extracted with Et_2O. The combined organic phases were washed twice with sat. $NaHCO_3$ solution and H_2O, and dried over $MgSO_4$. The solvent was evaporated in vacuum and 428 mg (84%) of product **11** was obtained as a yellowish solid and subjected to the next step without further purification. The spectroscopic data were in agreement with literature values [4].

R_f 0.33 (petrol ether/EtOAc = 1/1); ^{1}H NMR (500 MHz, $CDCl_3$, TMS) δ 3.29 (dd, J = 7.1, 0.6 Hz, 2H, CH_2), 3.73 (s, 3H, CH_3), 6.41 (dt, J = 15.9, 7.1 Hz, 1H, CH=C<u>H</u>CH_2), 6.53 (d, J = 15.9 Hz, 1H, C<u>H</u>=CHCH_2), 7.46 (d, J = 8.2 Hz, 2H, $C^{ar}H$), 7.56 (d, J = 8.2 Hz, 2H, $C^{ar}H$).

(4*R*,5*R*)-4-Hydroxy-5-[4-(trifluoromethyl)phenyl]dihydrofuran-2(3*H*)-one (12)

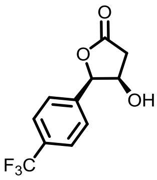

M = 246.18 g mol^{-1} $C_{11}H_9F_3O$

$K_2OsO_4 \cdot 2H_2O$ (17 mg, 0.047 mmol, 1 mol %), $K_3[Fe(CN)_6]$ (3.87 g, 11.77 mmol, 2.5 equiv) and K_2CO_3 (1.95 g, 14.13 mmol, 3.0 equiv) were dissolved in H_2O (20.5 mL). *tert*-Butanol (20.0 mL), $(DHQD)_2$-PHAL (36.5 mg, 0.047 mmol, 1 mol %) and methanesulfonamide (448 mg, 4.71 mmol, 1.0 equiv) were added. The mixture was cooled to 0 °C and methyl ester **11** (1.15 g, 4.71 mmol, 1.0 equiv), dissolved in *tert*-butanol (0.8 mL), was added. The reaction progress was monitored by TLC. After vigorous stirring for 42 h at 0 °C conversion was usually complete. Na_2SO_3 (5.84 g, 47.10 mmol, 10.0 equiv) and H_2O (10 mL) were added and the mixture was allowed to warm up to rt and stirred for a further 2 h at rt. After separation of the phases the aqueous layer was extracted with Et_2O (4 × 50 mL). The combined organic layers

were washed with H_2O (2 × 50 mL), dried over $MgSO_4$ and filtered through a plug of silica. The solvent was evaporated to dryness in vacuum, the residue was recrystallized (petrol ether/EtOAc = 10/1) and dried over several hours in vacuum to yield **12** (307 mg, 1.25 mmol, 27%, $[\alpha]_D^{21}$ = −29.7) as light beige crystals. The residue of the mother liquor was purified by flash chromatography (petrol ether/EtOAc = 3/2) to give **12** (214 mg, 0.87 mmol, 18%, $[\alpha]_D^{21}$ = −27.7) as a white solid. The product **12** was obtained in enantiomerically pure form as shown by chiral HPLC with a total yield of 521 mg (2.12 mmol, 45%).

R_f 0.21 (petrol ether/EtOAc = 1/1); $[\alpha]_D^{21}$ −29.7 (*c* 1.00 in MeOH); HPLC: t_R 12.1 min (method M4); ^{1}H NMR (500 MHz, $CDCl_3$, TMS) δ 1.72 (d, *J* = 1.5 Hz, 1H, OH), 2.73 (dm, *J* = 17.5 Hz, 1H, $C^{\alpha}\underline{H}^{A}H^{B}$), 2.93 (dd, *J* = 17.5, 5.0 Hz, 1H, $C^{\alpha}H^{A}\underline{H}^{B}$), 4.70 (m, 1H, $C^{\beta}H$), 5.54 (d, *J* = 3.5 Hz, 1H, $C^{\gamma}H$), 7.50 (d, *J* = 8.1 Hz, 2H, $C^{ar}H$), 7.70 (d, *J* = 8.1 Hz, 2H, $C^{ar}H$); ^{13}C NMR (126 MHz, $CDCl_3$, TMS) δ 38.6 ($C^{\alpha}H_2$), 70.1 ($C^{\beta}H$), 84.4 ($C^{\gamma}H$), 123.8 (q, *J* = 272.3 Hz, CF_3), 125.8 (q, *J* = 3.5 Hz, $\underline{C}^{ar}HCCF_3$), 126.8 ($C^{ar}H$), 131.1 (q, *J* = 32.6 Hz, $\underline{C}^{ar}CF_3$), 137.4 (C^{ar}), 175.0 (C=O); ^{19}F NMR (470 MHz, $CDCl_3$): δ −62.7 (s, 3F, CF_3); IR (ATR) ṽ (cm^{-1}) 3410 w, 2359 s, 2342 m, 2152 w, 2032 w, 1987 w, 1752 s, 1621 m, 1416 m, 1326 s, 1230 w, 1211 m, 1159 s, 1106 s, 1065 s, 1033 s, 1016 m, 987 m, 956 w, 901 w, 853 m, 792 s, 723 w, 703 m; HRMS–EI (m/z)$^-$: $[M + Cl]^-$ calcd for $[C_{11}H_9F_3O + Cl]^-$ 281.01978; found, 281.01894.

(4*S*,5*S*)-4-Hydroxy-5-[4-(trifluoromethyl)phenyl]dihydrofuran-2(3*H*)-one (*ent*-12)

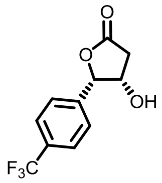

M = 246.18 g mol^{-1} $C_{11}H_9F_3O$

$K_2OsO_4\cdot 2H_2O$ (7.0 mg, 0.02 mmol, 1 mol %), $K_3[Fe(CN)_6]$ (1.55 g, 4.7 mmol, 2.5 equiv) and K_2CO_3 (0.78 g, 5.64 mmol, 3.0 equiv) were dissolved in H_2O (8.2 mL).

tert-Butanol (8.0 mL), $(DHQ)_2$-PHAL (15.0 mg, 0.02 mmol, 1 mol %) and methanesulfonamide (179 mg, 1.88 mmol, 1.0 equiv) were added. The mixture was cooled to 0 °C and methyl ester **11** (0.46 g, 1.88 mmol, 1.0 equiv), dissolved in *tert*-butanol (0.4 mL), was added. The reaction progress was monitored by TLC. After vigorous stirring for 42 h at 0 °C conversion was usually complete. Na_2SO_3 (2.37 g, 18.80 mmol, 10.0 equiv) and H_2O (4.0 mL) were added and the mixture was allowed to warm up to rt and stirred for further 2 h at rt.

After separation of the phases the aqueous layer was extracted with Et_2O (4 × 20 mL). The combined organic layers were washed with H_2O (2 × 25 mL), dried over $MgSO_4$ and filtered through a plug of silica. The solvent was evaporated to dryness in vacuum and the residue was purified by flash chromatography (petrol ether/EtOAc = 3/2). 226 mg (0.92 mmol, 49%) of the lactone *ent*-**12** were obtained enantiomerically pure as a colorless crystalline solid. $[\alpha]_D^{21}$ +31.1 (*c* 1.00 in MeOH); HPLC: t_R 16.2 min (method M4). The other physical data of *ent*-**12** were in agreement with those of the enantiomer **12**.

(3*R*,4*R*,5*R*)-4-Hydroxy-5-[4-(trifluoromethyl)phenyl]-3-methyldihydrofuran-2(3*H*)-one (13)

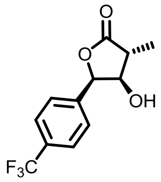

M = 260.21 g mol^{-1} $C_{12}H_{11}F_3O_3$

Diisopropylamine (0.45 mL, 3.05 mmol, 2.5 equiv) in abs. THF (7.6 mL) was cooled to −78 °C and *n*-BuLi (1.3 M in hexane, 2.21 mL, 3.05 mmol, 2.5 equiv) was added dropwise. The solution was stirred for 15 min at −78°C and then for 30 min without any cold bath. It was then cooled to −78 °C and **12** (300 mg, 1.22 mmol, 1.0 equiv), dissolved in abs. THF (5.1 mL), was added with a syringe pump over 90 min. The reaction mixture was stirred overnight at −78 °C, cooled to −90 °C and methyl iodide

(0.22 mL, 3.66 mmol, 3.0 equiv) was added with a syringe pump over 150 min at the same temperature. The reaction mixture was stirred for 3 d at −78 °C and then the reaction was quenched by addition of AcOH (0.12 mL) in abs. THF (0.25 mL). It was allowed to warm up to rt and the solvents were evaporated in vacuum (50 °C) to give a viscous, orange-brown suspension. This was transferred into a separating funnel with H_2O and Et_2O (10 mL each) and the phases were separated. The aqueous phase was extracted with Et_2O (4 × 10 mL). The combined organic phases were washed with 5% $KHSO_4$ solution (10 mL, w/v) and brine, and dried over $MgSO_4$, and the solvent was evaporated in vacuum. The residue was purified by column chromatography over silica gel (pentane/EtOAc = 1/1) to yield 154 mg (0.592 mmol, 48%) **13** as a light-brown solid in diastereomerically pure form according to ^{1}H NMR.

R_f 0.53 (pentane/EtOAc = 1/1); $[\alpha]_D^{21}$ −10.5 (*c* 1.00 in MeOH); ^{1}H NMR (500 MHz, $CDCl_3$) δ 1.41 (d, *J* = 7.7 Hz, 3H, CH_3), 1.58 (s, 1H, OH), 2.76, (dq, *J* = 7.7, 2.9 Hz, 1H, $C^{\alpha}H$), 4.36 (m, 1H, $C^{\beta}H$), 5.64 (d, *J* = 4.4 Hz, 1H, $C^{\gamma}H$), 7.50 (d, *J* = 8.1 Hz, 2H, $C^{ar}H$), 7.70 (d, *J* = 8.1 Hz, 2H, $C^{ar}H$); $^{13}C\{^1H\}$ NMR (126 MHz, $CDCl_3$) δ 13.0 (CH_3), 43.7 ($C^{\alpha}H$), 75.9 ($C^{\beta}H$), 81.8 ($C^{\gamma}H$), 123.8 (q, *J* = 272.3 Hz, CF_3), 125.8 (q, *J* = 3.5 Hz, $\underline{C}^{ar}HCCF_3$), 126.7 ($C^{ar}H$), 131.1 (q, *J* = 32.7 Hz, $\underline{C}^{ar}CF_3$), 137.5 (C^{ar}), 177.8 (C=O); ^{19}F NMR (470 MHz, $CDCl_3$) δ −62.7 (s, 3F, CF_3); IR (ATR) ṽ (cm^{-1}) 3735 w, 2360 vs, 2341 s, 1172 w, 1321 w, 1165 w, 1108 w, 1065 w, 999 w, 792 w, 669 m; HRMS–EI ($C_{12}H_{11}F_3O_3$): calcd 260.06603; found, 260.06390.

Methyl (*S*)-2-[(4*R*,5*R*)-5-(4-(trifluoromethyl)phenyl)-2,2-dimethyl-1,3-dioxolan-4-yl]propanoate (14)

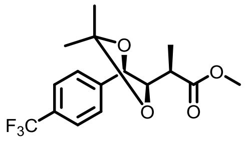

M = 332.31 g mol^{-1} $C_{16}H_{19}F_3O_4$

Abs. MeOH (0.26 mL) and abs. acetone dimethylketal (0.98 mL) were added to lactone **13** (206 mg, 0.793 mmol, 1.0 equiv) and Amberlyst-15® (16 mg). The reaction mixture was stirred for 8 d at rt, was then diluted with pentane and filtered through a pad of Celite. The residue was washed with pentane (200 mL) and the combined filtrates were washed with brine. After drying over $MgSO_4$ the solvent was evaporated in vacuum and the residue was purified by flash chromatography (pentane/EtOAc = 20/1) to yield 152 mg (2.12 mmol, 58%) **14** as a yellowish oil.

R_f 0.48 (pentane/EtOAc = 15/1); $[\alpha]_D^{21}$ −11.5 (*c* 1.00 in MeOH); ^{1}H NMR (500 MHz, $CDCl_3$) δ 1.28 (d, *J* = 7.0 Hz, 3H, $C^{\alpha}HC\underline{H}_3$), 1.48 (s, 3H, $C(CH_3)_2$), 1.56 (s, 3H, $C(CH_3)_2$), 2.70 (m, 1H, $C^{\alpha}H$), 3.44 (s, 3H, CO_2CH_3), 4.09 (dd, *J* = 8.2, 6.1 Hz, 1H, $C^{\beta}H$), 4.80 (d, *J* = 8.2 Hz, 1H, $C^{\gamma}H$), 7.49 (d, *J* = 8.0 Hz, 2H, $C^{ar}H$), 7.62 (d, *J* = 8.0 Hz, 2H, $C^{ar}H$); $^{13}C\{^1H\}$ NMR (126 MHz, $CDCl_3$) δ 12.9 ($C^{\alpha}H\underline{C}H_3$), 27.1 ($C(\underline{C}H_3)_2$), 27.2 ($C(\underline{C}H_3)_2$), 41.5 ($C^{\alpha}H$), 51.3 ($CO_2\underline{C}H_3$), 80.6 ($C^{\beta}H$), 83.3 ($C^{\gamma}H$), 109.5 ($\underline{C}(CH_3)_2$), 124.0 (q, *J* = 272.2 Hz, CF_3), 125.4 (q, *J* = 3.4 Hz, $\underline{C}^{ar}HCCF_3$), 127.6 ($C^{ar}H$), 130.7 (q, *J* = 32.7 Hz, $\underline{C}^{ar}CF_3$), 141.7 (C^{ar}), 173.6 (CO_2); ^{19}F NMR (470 MHz, $CDCl_3$) δ −62.7 (s, 3F, CF_3); IR (ATR) ṽ (cm^{-1}) 2987 w, 2941 w, 1735 s, 1621 w, 1456 m, 1435 w, 1422 w, 1372 m, 1322 s, 1236 m, 1162 m, 1162 m, 1121 m, 1063 s, 1016 s, 982 w, 888 m, 833 s, 812 m, 766 w; HRMS–EI (*m*/*z*)$^+$: [M + Na]$^+$ calcd for $[C_{16}H_{19}F_3O_4 + Na]^+$ 355.11276; found, 355.11285.

(2*S*,3*S*)-2-[(4*R*,5*R*)-5-(4-(Trifluoromethyl)phenyl)-2,2-dimethyl-1,3-dioxolan-4-yl]hex-5-en-3-ol (16)

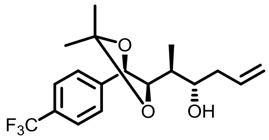

M = 344.37 g mol^{-1} $C_{18}H_{23}F_3O_3$

A solution of **14** (145 mg, 0.436 mmol, 1.0 equiv) in abs. CH_2Cl_2 (2.7 mL) was cooled to −78 °C and a solution of DIBAL-H (1.2 M in toluene, 0.84 mL, 1.00 mmol, 2.3 equiv) was added dropwise over 10 min. The reaction mixture was stirred for 4.5 h at the same temperature, and then cooled to −100 °C. The reaction was quenched by slow addition of abs. MeOH (0.55 mL). The solution was allowed to warm up to rt and diluted with 1 M AcOH (10 mL) and pentane (10 mL). After phase separation the aqueous phase was extracted with pentane (4 × 10 mL). The combined organic phases were washed with 1 M AcOH (6 mL) and brine (10 mL), dried over $MgSO_4$ and evaporated in vacuum. The crude aldehyde (*R*)-2-[(4*R*,5*R*)-5-(4-trifluoromethyl)phenyl)-2,2-dimethyl-1,3-dioxolan-4-yl]propanal (**15**) can be stored under argon atmosphere overnight at 5 °C. It was usually subjected to further conversions without purification.

Aldehyde **15** (0.436 mmol, 1.0 equiv) was dissolved in abs. CH_2Cl_2 (0.33 mL) and added dropwise at −78 °C to a solution of $MgBr_2 \cdot Et_2O$ (171 mg, 0.664 mmol, 1.5 equiv) in abs. CH_2Cl_2 (0.37 mL). The mixture was stirred for 15 min, in which time it turned red. Subsequently, allyl-tri-*n*-butylstannane (205 µL, 0.660 mmol, 1.5 equiv) in abs. CH_2Cl_2 (0.90 mL) was added dropwise and the reaction mixture was stirred overnight at −78 °C. Et_2O (15 mL) was added and the solution was allowed to warm to rt. H_2O (10 mL) was added and the phases were separated. The aqueous phase was extracted with Et_2O (3 × 15 mL) and the combined organic phases were washed with brine (10 mL), and dried over $MgSO_4$, and the solvent was evaporated in

vacuum. Flash chromatography (pentane/EtOAc = 12/1) and drying in high vacuum yielded 106 mg **16** (0.308 mmol, 71% over 2 steps) as a clear colorless oil.

R_f 0.26 (pentane/EtOAc = 10/1); $[\alpha]_D^{21}$ −1.5 (*c* 1.00 in MeOH); ^{1}H NMR (500 MHz, $CDCl_3$) δ 1.09 (d, *J* = 7.0 Hz, 3H, $CHC\underline{H}_3$), 1.50 (s, 3H, $C(CH_3)_2$), 1.56 (s, 3H, $C(CH_3)_2$), 1.75 (ddq, *J* = 7.0, 5.9, 2.0 Hz, 1H, $C\underline{H}CH_3$), 2.13 (m, 1H, $C\underline{H}^AH^BCH{=}CH_2$), 2.20 (br m, 1H, OH), 2.30 (m, 1H, $CH^A\underline{H}^BCH{=}CH_2$), 3.59 (m, 1H, $C\underline{H}OH$), 4.11 (dd, *J* = 8.8, 2.1 Hz, 1H, $C^{4'}H$), 4.83 (d, *J* = 8.8 Hz, 1H, $C^{5'}H$), 4.96–5.07 (m, 2H, $CH{=}C\underline{H}_2$), 5.72 (dddd, *J* = 16.9, 10.1, 7.7, 6.6 Hz, 1H, $C\underline{H}{=}CH_2$), 7.48 (d, *J* = 8.0 Hz, 2H, $C^{ar}H$), 7.62 (d, *J* = 8.0 Hz, 2H, $C^{ar}H$); $^{13}C\{^1H\}$ NMR (126 MHz, $CDCl_3$) δ 10.5 ($CH\underline{C}H_3$), 27.0 ($C(\underline{C}H_3)_2$), 27.1 ($C(\underline{C}H_3)_2$), 36.4 ($\underline{C}HCH_3$), 39.5 ($\underline{C}H_2CH{=}CH_2$), 73.2 (CHOH), 79.2 ($C^{5'}H$), 82.6 ($C^{4'}H$), 109.3 ($\underline{C}(CH_3)_2$), 118.1 ($CH{=}\underline{C}H_2$), 124.0 (q, *J* = 272.2 Hz, CF_3), 125.5 (q, *J* = 3.7 Hz, $\underline{C}^{ar}HCCF_3$), 126.9 ($C^{ar}H$), 130.4 (q, *J* = 32.4 Hz, $\underline{C}^{ar}CF_3$), 134.6 ($\underline{C}H{=}CH_2$), 142.1 (C^{ar}); ^{19}F NMR (470 MHz, $CDCl_3$) δ −62.6 (s, 3F, CF_3); IR (ATR) ṽ (cm^{-1}) 3453 br w, 2984 w, 2934 w, 1640 w, 1621 w, 1457 w, 1419 w, 1381 w, 1371 w, 1322 s, 1233 m, 1163 m, 1122 s, 1065 s, 1015 s, 994 m, 915 w, 887 m, 832 s, 767 w; HRMS–EI (*m*/*z*)$^-$: $[M + Cl]^-$ calcd for $[C_{18}H_{23}F_3O_3 + Cl]^-$ 379.12933; found, 379.12961.

Fragment condensation and diol-epoxide-transformation

H-uA[Acetonide]-C$^{4'}$-trifluoromethyl-uB-OTce (18)

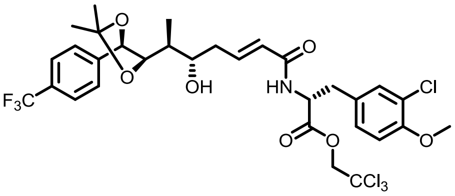

M = 731.41 g mol^{-1} $C_{31}H_{34}Cl_4F_3NO_7$

A mixture of the unit B building block **17** (154 mg, 0.366 mmol, 1.2 equiv) and Grubbs II catalyst (13 mg, 0.015 mmol, 5 mol %) was dried for 1 h under high vacuum. Under argon atmosphere it was dissolved in abs. CH_2Cl_2 (2.0 mL) and a

solution of unit A building block **16** (105 mg, 0.305 mmol, 1.0 equiv) in abs. CH_2Cl_2 (1.35 mL) was added at rt. The mixture was heated under reflux overnight in the dark and then allowed to cool to rt. The solvent was evaporated in vacuum (bath temperature ≤ 30 °C) and the residue was purified by flash chromatography (pentane/EtOAc = 2/1 → 1/1 → 6/4). Drying in high vacuum yielded 170 mg of a mixture from 145 mg (85%) of the cross-metathesis product **18** and 25 mg (15%) of the homo-coupling product **23**. The latter could be separated after the next reaction step.

R_f 0.24 (pentane/EtOAc = 6/4); ^{1}H NMR (500 MHz, $CDCl_3$) δ 1.04 (d, *J* = 6.9 Hz, 3H, uA-$C^{\varepsilon}HC\underline{H}_3$), 1.46 (s, 3H, uA-$C(CH_3)_2$), 1.51 (s, 3H, uA-$C(CH_3)_2$), 1.72 (m, 1H, uA-$C^{\varepsilon}H$), 2.23 (m, 1H, uA-$C^{\gamma}\underline{H}^AH^B$), 2.34 (m, 1H, uA-$C^{\gamma}\underline{H}^AH^B$), 2.36 (br m, 1H, uA-OH), 3.05 (dd, *J* = 14.2, 6.1 Hz, 1H, uB-$C^{\beta}\underline{H}^AH^B$), 3.13 (dd, *J* = 14.4, 5.8 Hz, 1H, uB-$C^{\beta}H^A\underline{H}^B$), 3.66 (br m, uA-$C^{\delta}H$), 3.83 (s, 3H, uB-$OCH_3$), 4.02 (dd, *J* = 8.9, 2.1 Hz, 1H, uA-$C^{\zeta}H$), 4.67 (d, *J* = 11.9 Hz, 1H, uB-$C\underline{H}^AH^BCCl_3$), 4.74 (d, *J* = 11.9 Hz, 1H, uB-$CH^A\underline{H}^BCCl_3$), 4.80 (d, *J* = 8.8 Hz, 1H, uA-$C^{\eta}H$), 4.99 (ddd, *J* = 7.7, 6.0, 5.9 Hz, 1H, uB-$C^{\alpha}H$), 5.75 (dm, *J* = 15.3 Hz, 1H, uA-$C^{\alpha}H$), 5.84 (d, *J* = 7.8 Hz, 1H, uB-NH), 6.79 (ddd, *J* = 15.1, 8.0, 7.9 Hz, 1H, uA-$C^{\beta}H$), 6.80 (d, *J* = 8.4 Hz, 1H, uB- $C^{5'}H$), 6.98 (dd, *J* = 8.4, 2.4 Hz, 1H, uB-$C^{6'}H$), 7.12 (d, *J* = 2.2 Hz, 1H, uB- $C^{2'}H$), 7.43 (d, *J* = 7.9 Hz, 2H, $C^{ar}H$), 7.57 (d, *J* = 7.9 Hz, 2H, $C^{ar}H$); $^{13}C\{^1H\}$ NMR (126 MHz, $CDCl_3$) δ 10.6 (uA-$C^{\varepsilon}H\underline{C}H_3$), 27.0 (uA-$C(\underline{C}H_3)_2$), 27.1 (uA-$C(\underline{C}H_3)_2$), 36.5 (uA-$C^{\gamma}H_2$), 36.9 (uA-$C^{\varepsilon}H$), 37.7 (uB-$C^{\beta}H_2$), 53.0 (uB-$C^{\alpha}H$), 56.1 (uB-$OCH_3$), 73.3 (uA-$C^{\delta}H$), 74.7 (uB-$\underline{C}H_2CCl_3$), 79.1 (uA-$C^{\eta}H$), 82.7 (uA-$C^{\zeta}H$), 94.2 (uB-$CCl_3$), 109.3 (uA-$\underline{C}(CH_3)_2$), 112.2 (uB-$C^{5'}H$), 122.5 (uB-$C^{3'}$), 124.0 (q, *J* = 272.0 Hz, CF_3), 125.2 (uA-$C^{\alpha}H$), 125.6 (q, *J* = 3.7 Hz, $\underline{C}^{ar}HCCF_3$), 126.9 (uA-$C^{ar}H$), 128.4 (uB-$C^{6'}H$), 128.5 (uB-$C^{1'}$), 130.5 (q, *J* = 32.4 Hz, $\underline{C}^{ar}CF_3$), 130.9 (uB-$C^{2'}H$), 141.9 (uA-C^{ar}), 142.3 (uA-$C^{\beta}H$), 154.2 (uB-$C^{4'}$), 165.0 (uA-C=O), 170.1 (uB-C=O); ^{19}F NMR (470 MHz, $CDCl_3$) δ −62.5 (s, 3F, CF_3); HRMS–

MALDI (m/z)$^+$: [M + Na]$^+$ calcd for [$C_{31}H_{34}Cl_4F_3NO_7$ + Na]$^+$ 752.09337; found, 752.09273.

***seco*-uA[Acetonide]-C$^{4'}$-trifluoromethyl-cryptophycin-52 (20)**

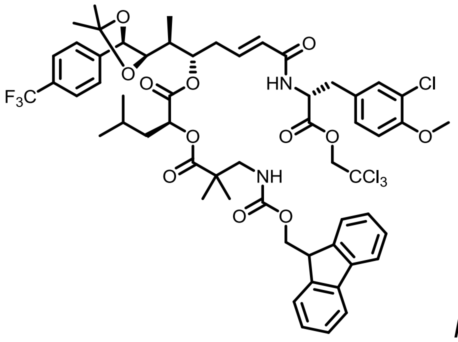

M = 1166.92 g mol^{-1} $C_{57}H_{63}Cl_4F_3N_2O_{12}$

The unit AB building block **18** (145 mg, 0.198 mmol, 1.0 equiv; in a mixture with 25 mg unit B homo-coupling product **23**) and unit CD building block **19** (135 mg, 0.296 mmol, 1.5 equiv) were esterified according to GP1. Purification by flash chromatography (pentane/EtOAc = 2/1) and drying in high vacuum yielded 151 mg (0.129 mmol, 65%) **20** as a colorless foam.

R_f 0.32 (pentane/EtOAc = 2/1); $[\alpha]_D^{21}$ −13.2 (c 1.00 in $CHCl_3$); ^{1}H NMR (500 MHz, $CDCl_3$) δ 0.89 (d, J = 6.2 Hz, 3H, uD-$C^{\delta}H_3$), 0.93 (d, J = 6.4 Hz, 3H, uD-$C^{\delta}H_3$), 1.13 (d, J = 7.5 Hz, 3H, uA-$C^{\varepsilon}HC\underline{H}_3$), 1.14 (s, 3H, uC-$C(CH_3)_2$), 1.22 (s, 3H, uC-$C(CH_3)_2$), 1.47 (s, 3H, uA-$C(CH_3)_2$), 1.49 (s, 3H, uA-$C(CH_3)_2$), 1.53 (m, 1H, uD-$C^{\beta}\underline{H}^AH^B$), 1.70–1.81 (m, 2H, uD-$C^{\beta}H^A\underline{H}^B$ and uD-$C^{\gamma}H$), 1.95 (m, 1H, uA-$C^{\varepsilon}H$), 2.46 (m, 1H, uA-$C^{\gamma}\underline{H}^AH^B$), 2.57 (m, 1H, uA-$C^{\gamma}H^A\underline{H}^B$), 3.04 (dd, J = 14.2, 6.7 Hz, 1H, uB-$C^{\beta}\underline{H}^AH^B$), 3.16 (dd, J = 14.2, 5.7 Hz, 1H, uB-$C^{\beta}H^A\underline{H}^B$), 3.38 (d, J = 6.6 Hz, 2H, uC-CH_2NH), 3.79–3.85 (m, 4H, uB-OCH_3 and uA-$C^{\zeta}H$), 4.21 (m, 1H, $C\underline{H}CH_2$, Fmoc), 4.30–4.37 (m, 2H, $CHC\underline{H}_2$, Fmoc), 4.64 (d, J = 11.9 Hz, 1H, uB-$C\underline{H}^AH^BCCl_3$), 4.76 (d, J = 11.9 Hz, 1H, uB-$CH^A\underline{H}^BCCl_3$), 4.76 (d, J = 8.7 Hz, 1H, uA-$C^{\eta}H$), 4.93 (dd, J = 9.9, 3.4 Hz, 1H, uD-$C^{\alpha}H$), 5.02 (m, 1H, uB-$C^{\alpha}H$), 5.08 (m, 1H, uA-$C^{\delta}H$), 5.78 (dm, J = 15.7 Hz, 1H, uA-$C^{\alpha}H$), 6.03 (m, 1H, NH), 6.41 (d, J = 8.0 Hz, 1H, NH), 6.71 (ddd, J = 15.5, 6.5, 6.5 Hz,

1H, uA-C$^{\beta}$H), 6.79 (d, J = 8.4 Hz, 1H, uB-C$^{5'}$H), 7.02 (dd, J = 8.4, 2.2 Hz, 1H, uB-C$^{6'}$H), 7.14 (d, J = 2.2 Hz, 1H, uB-C$^{2'}$H), 7.25–7.30 (m, 2H, C^{ar}H, Fmoc), 7.35–7.41 (m, 2H, C^{ar}H, Fmoc), 7.43 (d, J = 8.0 Hz, 2H, uA-C^{ar}H), 7.58 (d, J = 8.0 Hz, 2H, uA-C^{ar}H), 7.60–7.65 (m, 2H, C^{ar}H, Fmoc), 7.75 (d, J = 7.5 Hz, 2H, C^{ar}H, Fmoc); ^{13}C{^{1}H} NMR (126 MHz, $CDCl_3$) δ 9.2 (uA-C$^{\varepsilon}$H$\underline{C}$H$_3$), 21.4 (uD-C$^{\delta}$H$_3$), 22.2 (uC-C($\underline{C}$H$_3$)$_2$), 22.7 (uC-C($\underline{C}$H$_3$)$_2$), 23.1 (uD-C$^{\delta}$H$_3$), 24.9 (uD-C$^{\gamma}$H), 26.99 (uA-C($\underline{C}$H$_3$)$_2$), 27.01 (uA-C($\underline{C}$H$_3$)$_2$), 32.6 (uA-C$^{\gamma}$H$_2$), 35.5 (uA-C$^{\varepsilon}$H), 36.5 (uB-C$^{\beta}$H$_2$), 39.3 (uD-C$^{\beta}$H$_2$), 44.1 (uC-$\underline{C}$(CH$_3$)$_2$), 47.2 ($\underline{C}$HCH$_2$, Fmoc), 49.4 (uC-CH$_2$NH), 53.2 (uB-C$^{\alpha}$H), 56.0 (uB-OCH$_3$), 66.8 (CH$\underline{C}$H$_2$, Fmoc), 71.1 (uD-C$^{\alpha}$H), 74.6 (uB-$\underline{C}$H$_2$CCl$_3$), 75.7 (uA-C$^{\delta}$H), 79.3 (uA-C$^{\eta}$H), 81.9 (uA-C$^{\zeta}$H), 94.2 (uB-CCl$_3$), 109.5 (uA-$\underline{C}$(CH$_3$)$_2$), 112.0 (uB-C$^{5'}$H), 119.9 (C^{ar}H, Fmoc), 122.2 (uB-C$^{3'}$), 123.9 (q, J = 272.1 Hz, CF$_3$), 125.3 (C^{ar}H, Fmoc), 125.5 (uA-C$^{\alpha}$H), 125.7 (q, J = 3.7 Hz, $\underline{C}^{ar}$HCCF$_3$), 126.7 (uA-C^{ar}H), 127.6 (C^{ar}H, Fmoc), 128.4 (uB-C$^{6'}$H), 128.7 (uB-C$^{1'}$), 130.6 (q, J = 32.5 Hz, $\underline{C}^{ar}$CF$_3$), 131.1 (uB-C$^{2'}$H), 138.9 (uA-C$^{\beta}$H), 141.22, 141.24 (C^{ar}), 141.9 (uA-C^{ar}), 144.0 (C^{ar}), 154.0 (uB-C$^{4'}$), 156.9 (uC-NHCO$_2$), 165.1 (uA-C=O), 169.9 171.0, 176.7 (C=O) ; ^{19}F NMR (470 MHz, $CDCl_3$) δ −62.6 (s, 3F, CF$_3$); IR (ATR) ṽ (cm^{-1}) 3320 br w, 2960 br w, 2014 w, 1978 w, 1723 vs, 1676 m, 1644 m, 1521 m, 1502 s, 1449 m, 1371 w, 1323 s, 1255 br m, 1123 br s, 1065 s, 1015 br m, 889 w, 832 m, 809 m, 758 m, 740 m, 718 m; HRMS–MALDI (m/z)$^+$: [M + Na]$^+$ calcd for [$C_{57}H_{63}Cl_4F_3N_2O_{12}$ + Na]$^+$ 1187.29794; found, 1187.29628.

uA[Acetonide]-$C^{4'}$-trifluoromethyl-cryptophycin-52 (21)

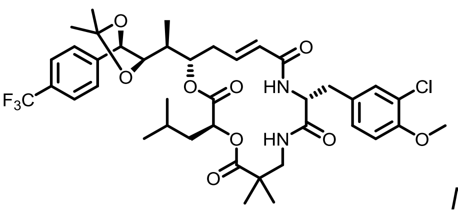

M = 795.28 g mol^{-1} $C_{40}H_{50}ClF_3N_2O_9$

Macrolactamization of **20** (150 mg, 0.129 mmol) according to GP2, purification by flash chromatography (pentane/EtOAc = 1/2), and drying in high vacuum yielded 84 mg (0.106 mmol, 82%) **21** as a colorless amorphous solid.

R_f 0.28 (pentane/EtOAc = 1/2); $[\alpha]_D^{21}$ +10.6 (c 0.93 in $CHCl_3$); ^{1}H NMR (500 MHz, $CDCl_3$) δ 0.78 (d, J = 6.6 Hz, 3H, uD-$C^{\delta}H_3$), 0.88 (d, J = 6.6 Hz, 3H, uD-$C^{\delta}H_3$), 1.14 (d, J = 6.3 Hz, 3H, uA-$C^{\varepsilon}HC\underline{H}_3$), 1.15 (s, 3H, uC-$C(CH_3)_2$), 1.21 (s, 3H, uC-$C(CH_3)_2$), 1.25 (ddd, J = 13.6, 9.4, 3.6 Hz, 1H, uD-$C^{\beta}\underline{H}^AH^B$), 1.47 (s, 3H, uA-$C(CH_3)_2$), 1.48 (s, 3H, uA-$C(CH_3)_2$), 1.62 (m, 1H, uD-$C^{\gamma}H$), 1.73 (ddd, J = 13.8, 10.5, 5.0 Hz, 1H, uD-$C^{\beta}H^A\underline{H}^B$), 1.83 (m, 1H, uA-$C^{\varepsilon}H$), 2.24 (ddd, J = 14.4, 11.0, 11.0 Hz, 1H, uA-$C^{\gamma}\underline{H}^AH^B$), 2.57 (dm, J = 14.6 Hz, 1H, uA-$C^{\gamma}H^A\underline{H}^B$), 3.03 (dd, J = 14.6, 7.6 Hz, 1H, uB-$C^{\beta}\underline{H}^AH^B$), 3.10 (dd, J = 14.6, 5.0 Hz, 1H, uB-$C^{\beta}H^A\underline{H}^B$), 3.13 (dd, J = 13.5, 3.6 Hz, 1H, uC-$C\underline{H}^AH^BNH$), 3.38 (dd, J = 13.5, 8.4 Hz, 1H, uC-$CH^A\underline{H}^BNH$), 3.77 (dd, J = 8.7, 1.6 Hz, 1H, uA-$C^{\zeta}H$), 3.87 (s, 3H, uB-OCH_3), 4.73 (ddd, J = 7.7, 5.1, 5.1 Hz, 1H, uB-$C^{\alpha}H$), 4.75 (d, J = 8.3 Hz, 1H, uA-$C^{\eta}H$), 4.76 (dd, J = 10.4, 3.6 Hz, 1H, uD-$C^{\alpha}H$), 5.04 (ddd, J = 11.4, 7.4, 2.1 Hz, 1H, uA-$C^{\delta}H$), 5.57 (d, J = 7.8 Hz, 1H, NH), 5.67 (dm, J = 15.1 Hz, 1H, uA-$C^{\alpha}H$), 6.69 (ddd, J = 15.1, 10.6, 4.3 Hz, 1H, uA-$C^{\beta}H$), 6.83 (d, J = 8.4 Hz, 1H, uB-$C^{5'}H$), 7.05 (dd, J = 8.4, 2.2 Hz, 1H, uB-$C^{6'}H$), 7.18 (m, 1H, NH), 7.19 (d, J = 2.1 Hz, 1H, uB-$C^{2'}H$), 7.45 (d, J = 8.0 Hz, 2H, uA-$C^{ar}H$), 7.63 (d, J = 8.0 Hz, 2H, uA-$C^{ar}H$); $^{13}C\{^1H\}$ NMR (126 MHz, $CDCl_3$) δ 9.5 (uA-$C^{\varepsilon}H\underline{C}H_3$), 21.2 (uD-$C^{\delta}H_3$), 22.7 (uC-$C(\underline{C}H_3)_2$), 22.8 (uC-$C(\underline{C}H_3)_2$), 22.9 (uD-$C^{\delta}H_3$), 24.6 (uD-$C^{\gamma}H$), 26.96 (uA-$C(\underline{C}H_3)_2$), 27.03 (uA-$C(\underline{C}H_3)_2$), 35.3 (uB-$C^{\beta}H_2$), 36.1 (uA-$C^{\gamma}H_2$), 36.5 (uA-$C^{\varepsilon}H$), 39.5 (uD-$C^{\beta}H_2$), 42.7 (uC-$\underline{C}(CH_3)_2$), 46.4 (uC-$CH_2NH$), 54.3 (uB-$C^{\alpha}H$), 56.1 (uB-$OCH_3$), 71.0 (uD-

$C^{\alpha}H$), 75.8 (uA-$C^{\delta}H$), 79.1 (uA-$C^{\eta}H$), 82.1 (uA-$C^{\zeta}H$), 109.7 (uA-$\underline{C}(CH_3)_2$), 112.3 (uB-$C^{5'}H$), 122.5 (uB-$C^{3'}$), 123.9 (q, J = 272.5 Hz, CF_3), 124.4 (uA-$C^{\alpha}H$), 125.7 (q, J = 3.5 Hz, $\underline{C}^{ar}HCCF_3$), 126.5 (uA-$C^{ar}H$), 128.2 (uB-$C^{6'}H$), 129.5 (uB-$C^{1'}$), 130.6 (q, J = 32.5 Hz, $\underline{C}^{ar}CF_3$), 130.9 (uB-$C^{2'}H$), 142.2 (uA-$C^{\beta}H$ and uA-C^{ar}), 154.0 (uB-$C^{4'}$), 165.0 (uA-C(O)NH), 170.1 (C(O)NH), 170.2, 177.9 (C=O); ^{19}F NMR (470 MHz, $CDCl_3$) δ −62.6 (s, 3F, CF_3); IR (ATR) ṽ (cm^{-1}) 3411 br w, 2961 br w, 1747 m, 1719 m, 1655 br m, 1503 s, 1472 w, 1370 w, 1324 s, 1257 w, 1125 br s, 1066 s, 1015 m, 969 w, 869 w, 832 w; HRMS–EI (m/z)$^+$: [M + Na]$^+$ calcd for $[C_{40}H_{50}ClF_3N_2O_9 + Na]^+$ 817.30491; found, 817.30490.

uA-$C^{4'}$-Trifluoromethyl-cryptophycin-52 (22)

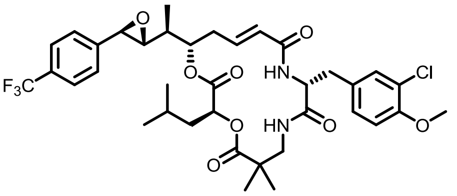

M = 737.20 g mol^{-1} $C_{37}H_{44}ClF_3N_2O_8$

The acetonide **21** (31 mg, 0.039 mmol) was converted into the epoxide according to GP3–6 and the crude product was purified by flash chromatography (pentane/EtOAc = 1/3). The purified product was dissolved in CH_3CN and H_2O and the mixture was lyophilized to give 11.3 mg (0.015 mmol, 39% over 4 steps) of epoxide **22** as voluminous, colorless solid.

R_f 0.35 (pentane/EtOAc = 1/3); $[\alpha]_D^{21}$ +16.8 (c 0.45 in $CHCl_3$); ^1H NMR (500 MHz, $CDCl_3$) δ 0.82 (d, J = 6.6 Hz, 3H, uD-$C^{\delta}H_3$), 0.85 (d, J = 6.6 Hz, 3H, uD-$C^{\delta}H_3$), 1.15 (d, J = 7.0 Hz, 3H, uA-$C^{\varepsilon}HC\underline{H}_3$), 1.16 (s, 3H, uC-$C(CH_3)_2$), 1.22 (s, 3H, uC-$C(CH_3)_2$), 1.30 (ddd, J = 13.9, 8.8, 3.6 Hz, 1H, uD-$C^{\beta}\underline{H}^AH^B$), 1.65 (m, 1H, uD-$C^{\gamma}H$), 1.73 (ddd, J = 14.6, 10.6, 4.9 Hz, 1H, uD-$C^{\beta}H^A\underline{H}^B$), 1.82 (m, 1H, uA-$C^{\varepsilon}H$), 2.43 (ddd, J = 14.5, 11.1, 11.0 Hz, 1H, uA-$C^{\gamma}\underline{H}^AH^B$), 2.57 (ddd, J = 14.5, 2.2, 2.2 Hz, 1H, uA-$C^{\gamma}H^A\underline{H}^B$), 2.90 (dd, J = 7.4, 1.9 Hz, 1H, uA-$C^{\zeta}H$), 3.03-3.13 (m, 3H, uB-$C^{\beta}\underline{H}^AH^B$, uB-$C^{\beta}H^A\underline{H}^B$

and uC-C$\underline{H}^{A}H^{B}$NH), 3.42 (dd, *J* = 13.5, 8.6 Hz, 1H, uC-CH$^{A}\underline{H}^{B}$NH), 3.74 (d, *J* = 1.9 Hz, 1H, uA-C$^{\eta}$H), 3.87 (s, 3H, uB-OCH$_3$), 4.74 (ddd, *J* = 7.5, 7.4, 5.3 Hz, 1H, uB-C$^{\alpha}$H), 4.82 (dd, *J* = 10.3, 3.5 Hz, 1H, uD-C$^{\alpha}$H), 5.20 (ddd, *J* = 11.3, 5.0, 2.0 Hz, 1H, uA-C$^{\delta}$H), 5.49 (d, *J* = 7.8 Hz, 1H, NH), 5.72 (dm, *J* = 15.1 Hz, 1H, uA-C$^{\alpha}$H), 6.76 (ddd, *J* = 14.9, 10.6, 4.2 Hz, 1H, uA-C$^{\beta}$H), 6.84 (d, *J* = 8.3 Hz, 1H, uB-C$^{5'}$H), 7.04 (dd, *J* = 8.5, 2.2 Hz, 1H, uB-C$^{6'}$H), 7.19 (d, *J* = 2.3 Hz, 1H, uB-C$^{2'}$H), 7.20 (m, 1H, NH), 7.37 (d, *J* = 8.0 Hz, 2H, uA-C^{ar}H), 7.63 (d, *J* = 8.0 Hz, 2H, uA-C^{ar}H); ^{13}C NMR (126 MHz, CDCl$_3$) δ 13.5 (uA-C$^{\varepsilon}$H$\underline{C}$H$_3$), 21.3 (uD-C$^{\delta}$H$_3$), 22.7 (uC- C($\underline{C}$H$_3$)$_2$), 22.8 (uC-C($\underline{C}$H$_3$)$_2$), 22.9 (uD-C$^{\delta}$H$_3$), 24.6 (uD-C$^{\gamma}$H), 35.3 (uB-C$^{\beta}$H$_2$), 36.9 (uA-C$^{\gamma}$H$_2$), 39.4 (uD-C$^{\beta}$H$_2$), 40.5 (uA-C$^{\varepsilon}$H), 42.7 (uC-$\underline{C}$(CH$_3$)$_2$), 46.4 (uC-CH$_2$NH), 54.3 (uB-C$^{\alpha}$H), 56.1 (uB-OCH$_3$), 58.2 (uA-C$^{\eta}$H), 63.5 (uA-C$^{\zeta}$H), 71.1 (uD-C$^{\alpha}$H), 75.7 (uA-C$^{\delta}$H), 112.3 (uB-C$^{5'}$H), 122.6 (uB-C$^{3'}$), 123.9 (q, *J* = 271.8 Hz, CF$_3$), 124.7 (uA-C$^{\alpha}$H), 125.7 (q, *J* = 3.6 Hz, $\underline{C}^{ar}$HCCF$_3$), 125.8 (uA-C^{ar}H), 128.3 (uB-C$^{6'}$H), 129.3 (uB-C$^{1'}$), 130.7 (q, *J* = 32.7 Hz, $\underline{C}^{ar}$CF$_3$), 130.9 (uB-C$^{2'}$H), 140.9 (uA-C^{ar}), 141.6 (uA-C$^{\beta}$H), 154.1 (uB-C$^{4'}$), 164.8 (uA-C(O)NH), 170.2 (C(O)NH), 170.4, 178.0 (C=O); ^{19}F NMR (470 MHz, CDCl$_3$, TMS) δ −62.6 (s, 3F, CF$_3$); IR (ATR) ṽ (cm^{-1}) 3851 w, 3733 w, 3674 w, 3415 w, 3272 w, 2958 br w, 2167 w, 1970 w, 1748 m, 1718 m, 1654 br m, 1504 br s, 1472 m, 1324 s, 1257 m, 1126 br s, 1066 s, 1017 m, 970 w, 894 w, 838 m; HRMS–EI (*m*/*z*)$^+$: [M + Na]$^+$ calcd for [$C_{37}H_{44}ClF_3N_2O_8$ + Na]$^+$ 759.26305; found, 759.26191.

3.2 Synthesis of the pentafluorophenyl-substituted unit B building block

(*R*)-2,2,2-Trichloroethyl 2-(*tert*-butoxycarbonylamino)-3-(pentafluorophenyl)-propanoate (25)

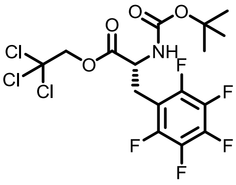

M = 486.65 g mol^{-1} $C_{16}H_{15}Cl_3F_5NO_4$

By analogy to Nahrwold [5] Boc-pentafluoro-D-phenylalanine (**24**) (1.00 g, 2.82 mmol, 1.00 equiv) was dissolved in abs. CH_2Cl_2 (14 mL), and abs. pyridine (0.46 mL, 5.63 mmol, 2.00 equiv) and trichloroethanol (0.41 mL, 4.22 mmol, 1.50 equiv) were added sequentially at rt. Then, the reaction mixture was cooled to 0 °C, and DCC (612 mg, 2.96 mmol, 1.05 equiv), dried for 30 min in high vacuum and suspended in abs. CH_2Cl_2 (1.0 mL), was added dropwise at 0 °C to the reaction mixture. The suspension was stirred overnight at the same temperature. The reaction was monitored by TLC. If the conversion was not complete, abs. pyridine (0.46 mL, 5.63 mmol, 2.00 equiv), trichloroethanol (0.41 mL, 4.22 mmol, 1.50 equiv) and solid DCC (260 mg, 1.27 mmol, 0.45 equiv) were added at 0 °C. The mixture was stirred for a further 5.5 h at 0 °C. The solids were removed by filtration and the residue was washed with CH_2Cl_2 (4 × 5 mL). The combined organic phases were washed with brine (10 mL), and dried over $MgSO_4$, and the solvent was evaporated to dryness in vacuum. The resulting white solid was purified by flash chromatography (petrol ether/EtOAc = 15/1) to yield 1.14 g (2.34 mmol, 83%) of product **25** as a colorless solid.

R_f 0.32 (hexane/EtOAc = 10/1); HPLC: t_R 5.6 min (method M1); $[\alpha]_D^{21}$ −7.8 (c 1.00 in $CHCl_3$); ^1H NMR (500 MHz, $CDCl_3$, TMS) δ 1.40 (s, 9H, $C(CH_3)_3$), 3.12 (dd, J = 14.1, 8.2 Hz, 1H, $C^{\beta}\underline{H}^{A}H^{B}$), 3.42 (dd, J = 14.1, 5.0 Hz, 1H, $C^{\beta}H^{A}\underline{H}^{B}$), 4.73 (ddd, J = 8.4, 5.2, 5.1 Hz, 1H, $C^{\alpha}H$), 4.79 (d, J = 12.0 Hz, 1H, $C\underline{H}^{A}H^{B}CCl_3$), 4.82 (d, J = 12.0 Hz, 1H,

$CH^AH^BCCl_3$), 5.10 (d, *J* = 8.5 Hz, 1H, NH); ^{13}C NMR (126 MHz, $CDCl_3$, TMS) δ 25.9 ($C^{\beta}H_2$), 28.1 ($C(\underline{C}H_3)_3$), 52.6 ($C^{\alpha}H$), 74.8 ($\underline{C}H_2CCl_3$), 80.7 ($\underline{C}(CH_3)_3$), 94.1 (CCl_3), 109.6 (tm, *J* = 18.2 Hz, $C_6F_5\underline{C}^{ipso}$), 137.4 (dm, *J* = 251.3 Hz, $C_6F_5\underline{C}^{meta}$), 140.5 (dm, *J* = 251.3 Hz, $C_6F_5\underline{C}^{para}$), 145.5 (dm, *J* = 246.2 Hz, $C_6F_5\underline{C}^{ortho}$), 154.8 ($NCO_2$), 169.6 ($\underline{C}O_2CH_2$); ^{19}F NMR (470 MHz, $CDCl_3$, TMS) δ −142.3 (dd, *J* = 22.4, 8.2 Hz, 2F, F^{ortho}), −155.0 (t, *J* = 20.8 Hz, 1F, F^{para}), −161.9 (td, *J* = 21.6, 8.2 Hz, 2F, F^{meta}); IR (ATR) ṽ (cm^{-1}) 3481 w, 2357 w, 2341 w, 1763 m, 1693 s, 1523 s, 1501 s, 1444 w, 1369 m, 1270 m, 1250 w, 1157 s, 1123 w, 1061 m, 959 s, 889 w, 826 w, 776 m, 720 s; HRMS–EI $(m/z)^+$: $[M + Na]^+$ calcd for $[C_{16}H_{15}Cl_3F_5NO_4 + Na]^+$ 507.98790; found, 507.98737.

(*R*)-2,2,2-Trichloroethyl 2-acrylamido-3-(pentafluorophenyl)propanoate (26)

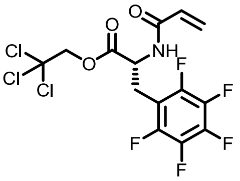

M = 440.58 g mol^{-1} $C_{14}H_9Cl_3F_5NO_3$

Adapting a literature procedure [5], a solution of **25** (728 mg, 1.50 mmol, 1.0 equiv) in TFA (1.46 mL) was stirred for 2 h at rt. Then the volatiles were evaporated in vacuum to dryness. The residue was co-evaporated twice with toluene, dissolved in CH_2Cl_2 (18 mL) and washed with saturated $NaHCO_3$ solution (10 mL). The organic phase was dried over $MgSO_4$ and evaporated in vacuum to dryness. The residue was dried overnight under high vacuum and dissolved in abs. CH_2Cl_2 (10 mL). The solution was cooled to 0 °C, and abs. NEt_3 (0.83 mL, 5.98 mmol, 4.0 equiv) and acryloyl chloride (278 µL, 3.74 mmol, 2.5 equiv) were added. The reaction mixture was stirred for 7 h in the dark at 0 °C. Then, EtOAc (47 mL) and 5% $KHSO_4$ solution (10 mL, w/v) were added, and the phases were separated. After phase separation, the organic phase was washed with saturated $NaHCO_3$ solution and brine (10 mL each), dried over

$MgSO_4$, and evaporated in vacuum to dryness. Purification by flash chromatography (CH_2Cl_2/Et_2O = 25/1) yielded 373 mg (0.847 mmol, 57%) of **26** as a colorless solid.

R_f 0.25 (hexane/EtOAc = 4/1); $[\alpha]_D^{21}$ −29.6 (*c* 1.00 in $CHCl_3$); ^1H NMR (500 MHz, $CDCl_3$, TMS) δ 3.19 (dd, *J* = 14.2, 7.7 Hz, 1H, $C^\beta\underline{H}^AH^B$), 3.48 (dd, *J* = 14.2, 5.3 Hz, 1H, $C^\beta H^A\underline{H}^B$), 4.80 (d, *J* = 11.9 Hz, 1H, $C\underline{H}^AH^BCCl_3$), 4.83 (d, *J* = 11.9 Hz, 1H, $CH^A\underline{H}^BCCl_3$), 5.15 (ddd, *J* = 7.9, 5.4, 5.3 Hz, 1H, $C^\alpha H$), 5.73 (dd, *J* = 10.4, 1.2 Hz, 1H, $CH{=}C\underline{H_2}^{trans}$), 6.08 (m, 1H, NH), 6.10 (dd, *J* = 16.9, 10.4 Hz, 1H, $C\underline{H}{=}CH_2$), 6.28 (dd, *J* = 17.0, 1.2 Hz, 1H, $CH{=}C\underline{H_2}^{cis}$); ^{13}C NMR (126 MHz, $CDCl_3$, TMS) δ 25.6 ($C^\beta H_2$), 51.2 ($C^\alpha H$), 75.0 ($\underline{C}H_2CCl_3$), 94.0 (CCl_3), 109.3 (tm, *J* = 18.2 Hz, $C_6F_5\underline{C}^{ipso}$), 128.2 ($CH{=}\underline{C}H_2$), 129.5 ($\underline{C}H{=}CH_2$), 137.4 (dm, *J* = 252.4 Hz, $C_6F_5\underline{C}^{meta}$), 140.6 (dm, *J* = 254.4 Hz, $C_6F_5\underline{C}^{para}$), 145.5 (dm, *J* = 245.9 Hz, $C_6F_5\underline{C}^{ortho}$), 165.2 ($NCO_2$), 169.3 ($\underline{C}O_2CH_2$); ^{19}F NMR (470 MHz, $CDCl_3$, TMS) δ −142.3 (dd, *J* = 21.9, 7.7 Hz, 2F, F^{ortho}), −154.3 (t, *J* = 20.8 Hz, 1F, F^{para}), −161.5 (td, *J* = 21.9, 8.1 Hz, 2F, F^{meta}); IR (ATR) ṽ (cm^{-1}) 3339 w, 2358 m, 2340 m, 1739 s, 1660 m, 1629 w, 1519 s, 1502 s, 1449 w, 1374 w, 1311 m, 1218 m, 1190 m, 1126 w, 1051 m, 1023 w, 983 m, 956 s, 827 w, 807 s, 785 m, 720 s; HRMS–MALDI $(m/z)^+$: $[M + Na]^+$ calcd for $[C_{14}H_9Cl_3F_5NO_3 + Na]^+$ 461.94604; found, 461.94609.

Fragment condensation and diol-epoxide-transformation

H-uA[Acetonide]-uB-pentafluorophenyl-OTce (28)

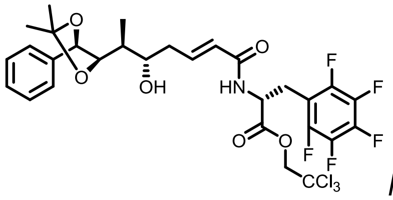

M = 688.90 g mol^{-1} $C_{29}H_{29}Cl_3F_5NO_6$

A mixture of the unit B building block **26** (100 mg, 0.227 mmol, 1.0 equiv) and Grubbs II catalyst (10.5 mg, 0.015 mmol, 5 mol %) was dissolved in abs. CH_2Cl_2 (1.50 mL) and stirred for 45 min at rt. Then, the unit A building block **27** (75 mg,

0.272 mmol, 1.2 equiv), dissolved in abs. CH_2Cl_2 (0.75 mL), was added and the mixture was heated under reflux overnight in the dark. After cooling to rt the solvent was evaporated in vacuum and the residue was purified by chromatography (pentane/EtOAc = 3/1) to yield 67 mg (0.097 mmol, 43%) of **28** as a brownish viscous oil.

R_f 0.56 (pentane/EtOAc = 2/1); $[\alpha]_D^{21}$ −20.6 (*c* 0.98 in $CHCl_3$); ^{1}H NMR (500 MHz, $CDCl_3$) δ 1.06 (d, *J* = 7.0 Hz, 3H, uA-$C^{\varepsilon}HC\underline{H}_3$), 1.49 (s, 3H, uA-$C(CH_3)_2$), 1.56 (s, 3H, uA-$C(CH_3)_2$), 1.77 (m, 1H, uA-$C^{\varepsilon}H$), 2.23–2.36 (m, 2H, uA-$C^{\gamma}H_2$), 2.53 (br m, 1H, uA-OH), 3.15 (dd, *J* = 14.1, 7.6 Hz, 1H, uB-$C^{\beta}\underline{H}^AH^B$), 3.42 (dd, *J* = 14.3, 5.6 Hz, 1H, uB-$C^{\beta}H^A\underline{H}^B$), 3.69 (br m, uA-$C^{\delta}H$), 4.05 (dd, *J* = 9.0, 2.3 Hz, 1H, uA-$C^{\zeta}H$), 4.78 (d, *J* = 11.9 Hz, 1H, uB-$C\underline{H}^AH^BCCl_3$), 4.79 (d, *J* = 8.5 Hz, 1H, uA-$C^{\eta}H$), 4.81 (d, *J* = 11.9 Hz, 1H, uB-$CH^A\underline{H}^BCCl_3$), 5.08 (ddd, *J* = 7.8, 5.5, 5.5 Hz, 1H, uB-$C^{\alpha}H$), 5.67 (dm, *J* = 15.4 Hz, 1H, uA-$C^{\alpha}H$), 5.95 (d, *J* = 8.1 Hz, 1H, uB-NH), 6.76 (ddd, *J* = 14.9, 7.4, 7.3 Hz, 1H, uA-$C^{\beta}H$), 7.32–7.38 (m, 5H, uA-$C^{ar}H$); ^{13}C NMR (126 MHz, $CDCl_3$) δ 10.9 (uA-$C^{\varepsilon}H\underline{C}H_3$), 25.7 (uB-$C^{\beta}H_2$), 27.0 (uA-$C(\underline{C}H_3)_2$), 27.2 (uA-$C(\underline{C}H_3)_2$), 36.5 (uA-$C^{\varepsilon}H$), 37.6 (uA-$C^{\gamma}H_2$), 51.2 (uB-$C^{\alpha}H$), 73.5 (uA-$C^{\delta}H$), 74.9 (uB-$\underline{C}H_2CCl_3$), 79.9 (uA-$C^{\eta}H$), 82.7 (uA-$C^{\zeta}H$), 94.0 (uB-$CCl_3$), 109.0 (uA-$\underline{C}(CH_3)_2$), 109.4 (tm, *J* = 18.2 Hz, $C_6F_5\underline{C}^{ipso}$), 124.7 (uA-$C^{\alpha}H$), 126.8, 128.5, 128.7 (uA-$C^{ar}H$), 137.4 (uA-C^{ar}), 137.4 (dm, *J* = 252.5 Hz, $C_6F_5\underline{C}^{meta}$), 140.5 (dm, *J* = 254.8 Hz, $C_6F_5\underline{C}^{para}$), 142.8 (uA-$C^{\beta}H$), 145.5 (dm, *J* = 245.9 Hz, $C_6F_5\underline{C}^{ortho}$), 165.2 (uA-C=O), 169.4 (uB-C=O); ^{19}F NMR (470 MHz, $CDCl_3$) δ −142.2 (dd, *J* = 22.5, 8.0 Hz, 2F, F^{ortho}), −154.5 (t, *J* = 20.9 Hz, 1F, F^{para}), −161.6 (td, *J* = 21.6, 7.9 Hz, 2F, F^{meta}); IR (ATR) ṽ (cm^{-1}) 2983 w, 2935 w, 2359 vs, 2341 s, 1736 m, 1670 m, 1637 m, 1520 s, 1503 s, 1453 w, 1371 m, 1235 m, 1167 m, 1123 w, 1040 m, 970 m, 882 w, 811 w, 755 m; HRMS–EI $(m/z)^+$: $[M + Na]^+$ calcd for $[C_{29}H_{29}Cl_3F_5NO_6 + Na]^+$ 710.08728; found, 710.08810; $[M + K]^+$ calcd for $[C_{29}H_{29}Cl_3F_5NO_6 + K]^+$ 726.06122; found, 726.06204; $[2 M + Na]^+$ calcd for $[(C_{29}H_{29}Cl_3F_5NO_6)_2 + Na]^+$ 1397.18534; found, 1397.18624.

seco-uA[Acetonide]-uB-pentafluorophenyl-cryptophycin-52 (29)

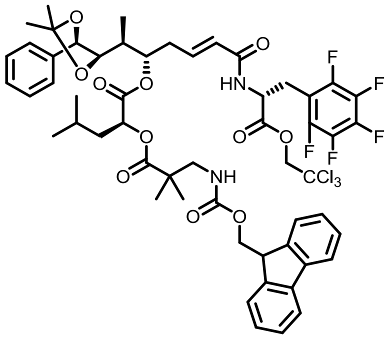

M = 1124.41g mol^{-1} $C_{55}H_{58}Cl_3F_5N_2O_{11}$

The unit AB building block **28** (67 mg, 97 µmol, 1.0 equiv) and unit CD building block **19** (66 mg, 146 µmol, 1.5 equiv) were esterified according to GP1. Purification by flash chromatography (pentane/EtOAc = 4/1), preparative RP-HPLC (method M3) and lyophilization yielded 49 mg (43.6 µmol, 45%) of **29** as a voluminous colorless solid.

R_f 0.24 (pentane/EtOAc = 4/1); $[\alpha]_D^{21}$ −9.0 (*c* 0.95 in $CHCl_3$); ^{1}H NMR (500 MHz, $CDCl_3$) δ 0.92 (d, *J* = 6.3 Hz, 3H, uD-$C^{\delta}H_3$), 0.97 (d, *J* = 6.2 Hz, 3H, uD-$C^{\delta}H_3$), 1.12 (d, *J* = 6.9 Hz, 3H, uA-$C^{\varepsilon}HC\underline{H}_3$), 1.16 (s, 3H, uC-$C(CH_3)_2$), 1.24 (s, 3H, uC-$C(CH_3)_2$), 1.45 (s, 3H, uA-$C(CH_3)_2$), 1.51 (s, 3H, uA-$C(CH_3)_2$), 1.58 (m, 1H, uD-$C^{\gamma}H$), 1.67–1.83 (m, 2H, uD-$C^{\beta}H_2$), 1.94 (m, 1H, uA-$C^{\varepsilon}H$), 2.35–2.52 (m, 2H, uA-$C^{\gamma}H_2$), 3.14 (dd, *J* = 14.2, 7.6 Hz, 1H, uB-$C^{\beta}\underline{H}^AH^B$), 3.36–45 (m, 3H, uB-$C^{\beta}H^A\underline{H}^B$ and uC-CH_2NH), 3.82 (dd, *J* = 8.8, 2.5 Hz, 1H, uA-$C^{\zeta}H$), 4.21 (m, 1H, $C\underline{H}CH_2$, Fmoc), 4.30–4.39 (m, 2H, $CHC\underline{H}_2$, Fmoc), 4.70 (d, *J* = 8.7 Hz, 1H, uA-$C^{\eta}H$), 4.72 (d, *J* = 11.9 Hz, 1H, uB-$C\underline{H}^AH^BCCl_3$), 4.74 (d, *J* = 11.9 Hz, 1H, uB-$CH^A\underline{H}^BCCl_3$), 4.89 (dd, *J* = 10.0, 3.3 Hz, 1H, uD-$C^{\alpha}H$), 5.02-5.15 (m, 2H, uB-$C^{\alpha}H$ and uA-$C^{\delta}H$), 5.68 (dm, *J* = 15.5 Hz, 1H, uA-$C^{\alpha}H$), 6.07 (m, 1H, NH), 6.61 (ddd, *J* = 15.4, 6.4, 6.4 Hz, 1H, uA-$C^{\beta}H$), 6.69 (d, *J* = 8.4 Hz, 1H, NH), 7.24–7.43 (m, 9H, uA-$C^{ar}H$ and $C^{ar}H$, Fmoc), 7.55–7.65 (m, 2H, $C^{ar}H$, Fmoc), 7.75 (d, *J* = 7.7 Hz, 2H, $C^{ar}H$, Fmoc); $^{13}C\{^1H\}$ NMR (126 MHz, $CDCl_3$) δ 9.4 (uA-$C^{\varepsilon}H\underline{C}H_3$), 21.3 (uD-$C^{\delta}H_3$), 22.1 (uC-$C(\underline{C}H_3)_2$), 22.8 (uC-$C(\underline{C}H_3)_2$), 23.1 (uD-

$C^{\delta}H_3$), 24.9 (uD-$C^{\gamma}H$), 25.4 (uB-$C^{\beta}H_2$), 27.0 (uA-C($\underline{C}H_3)_2$), 27.2 (uA-C($\underline{C}H_3)_2$), 32.4 (uA-$C^{\gamma}H_2$), 35.8 (uA-$C^{\varepsilon}H$), 39.2 (uD-$C^{\beta}H_2$), 44.1 (uC-$\underline{C}(CH_3)_2$), 47.2 ($\underline{C}HCH_2$, Fmoc), 49.5 (uC-CH_2NH), 51.2 (uB-$C^{\alpha}H$), 66.9 ($CH\underline{C}H_2$, Fmoc), 71.3 (uD-$C^{\alpha}H$), 74.8 (uB-$\underline{C}H_2CCl_3$), 75.6 (uA-$C^{\delta}H$), 80.3 (uA-$C^{\eta}H$), 82.1 (uA-$C^{\zeta}H$), 94.1 (uB-CCl_3), 109.0 (uA-$\underline{C}(CH_3)_2$), 119.9 ($C^{ar}H$, Fmoc), 124.9 (uA-$C^{\alpha}H$), 125.2 ($C^{ar}H$, Fmoc), 126.7 ($C^{ar}H$), 126.96, 127.00, 127.6 ($C^{ar}H$, Fmoc), 128.5, 128.7 ($C^{ar}H$), 137.3 (uA-C^{ar}), 139.5 (uA-$C^{\beta}H$), 141.23, 141.25, 143.96, 144.02 (C^{ar}), 157.0 (uC-$NHCO_2$), 165.8 (uA-C=O), 169.8, 171.0, 176.9 (C=O). The signals of the C_6F_5 group were not visible in the ^{13}C NMR spectrum because of their multiplicity and low intensity. ^{19}F NMR (470 MHz, $CDCl_3$) δ −142.1 (dd, J = 22.9, 7.9 Hz, 2F, F^{ortho}), −154.9 (t, J = 20.9 Hz, 1F, F^{para}), −161.9 (td, J = 22.0, 8.0 Hz, 2F, F^{meta}); IR (ATR) ṽ (cm^{-1}) 3368 br w, 2959 br m, 2249 w, 1721 br s, 1678 m, 1646 m, 1519 s, 1504 s, 1449 m, 1371 m, 1241 m, 1141 m, 1124 m, 1041 m, 1023 w, 969 m, 906 s, 813 w, 757 m, 726 s; HRMS–MALDI (m/z)$^+$: $[M + Na]^+$ calcd for $[C_{55}H_{58}Cl_3F_5N_2O_{11} + Na]^+$ 1145.29186; found, 1145.29277.

uA[Acetonide]-uB-pentafluorophenyl-cryptophycin-52 (30)

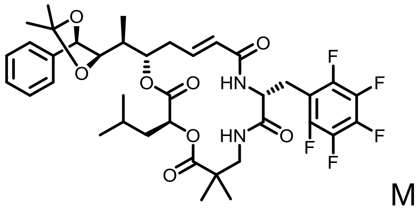

M = 752.76 g mol^{-1} $C_{38}H_{45}F_5N_2O_8$

Macrolactamization of **29** (48 mg, 42.7 µmol) according to GP2, purification by flash chromatography (pentane/EtOAc = 2/1) and drying in high vacuum yielded 26.5 mg (35.2 µmol, 83%) of **30** as a colorless crystalline solid.

R_f 0.21 (pentane/EtOAc = 2/1); $[\alpha]_D^{21}$ +21.4 (c 0.98 in $CHCl_3$); ^1H NMR (500 MHz, $CDCl_3$) δ 0.84 (d, J = 6.5 Hz, 3H, uD-$C^{\delta}H_3$), 0.93 (d, J = 6.6 Hz, 3H, uD-$C^{\delta}H_3$), 1.14 (d, J = 6.9 Hz, 3H, uA-$C^{\varepsilon}HC\underline{H}_3$), 1.18 (s, 3H, uC-$C(CH_3)_2$), 1.24 (s, 3H, uC-$C(CH_3)_2$),

1.36 (ddd, *J* = 13.8, 8.9, 3.5 Hz, 1H, uD-$C^{\beta}\underline{H}^{A}H^{B}$), 1.46 (s, 3H, uA-$C(CH_3)_2$), 1.50 (s, 3H, uA-$C(CH_3)_2$), 1.66 (m, 1H, uD-$C^{\gamma}H$), 1.75 (ddd, *J* = 13.9, 10.5, 5.0 Hz, 1H, uD-$C^{\beta}H^{A}\underline{H}^{B}$), 1.85 (m, 1H, uA-$C^{\varepsilon}H$), 2.22 (ddd, *J* = 14.3, 10.8, 10.8 Hz, 1H, uA-$C^{\gamma}\underline{H}^{A}H^{B}$), 2.47 (dm, *J* = 14.2 Hz, 1H, uA-$C^{\gamma}H^{A}\underline{H}^{B}$), 2.97 (dd, *J* = 14.5, 11.2 Hz, 1H, uB-$C^{\beta}\underline{H}^{A}H^{B}$), 3.20 (dd, *J* = 13.5, 6.7 Hz, 1H, uC-$C\underline{H}^{A}H^{B}NH$), 3.38 (dd, *J* = 13.5, 5.1 Hz, 1H, uC-$CH^{A}\underline{H}^{B}NH$), 3.44 (dd, *J* = 14.5, 4.3 Hz, 1H, uB-$C^{\beta}H^{A}\underline{H}^{B}$), 3.79 (dd, *J* = 8.8, 2.3 Hz, 1H, uA-$C^{\zeta}H$), 4.68 (m, 1H, uB-$C^{\alpha}H$), 4.71 (d, *J* = 8.7 Hz, 1H, uA-$C^{\eta}H$), 4.83 (dd, *J* = 10.4, 3.5 Hz, 1H, uD-$C^{\alpha}H$), 5.02 (ddd, *J* = 11.3, 7.2, 2.0 Hz, 1H, uA-$C^{\delta}H$), 5.68 (dm, *J* = 15.2 Hz, 1H, uA-$C^{\alpha}H$), 5.80 (m, 1H, NH), 6.61 (ddd, *J* = 15.1, 10.2, 4.8 Hz, 1H, uA-$C^{\beta}H$), 7.09 (m, 1H, NH), 7.32–7.41 (m, 5H, uA-$C^{ar}H$); $^{13}C\{^{1}H\}$ NMR (126 MHz, $CDCl_3$) δ 9.7 (uA-$C^{\varepsilon}H\underline{C}H_3$), 21.3 (uD-$C^{\delta}H_3$), 22.8 (uC- $C(\underline{C}H_3)_2$), 23.0 (uC-$C(\underline{C}H_3)_2$ and uD-$C^{\delta}H_3$), 24.0 (uB-$C^{\beta}H_2$), 24.7 (uD-$C^{\gamma}H$), 27.0 (uA-$C(\underline{C}H_3)_2$), 27.2 (uA-$C(\underline{C}H_3)_2$), 35.8 (uA-$C^{\gamma}H_2$), 36.7 (uA-$C^{\varepsilon}H$), 39.5 (uD-$C^{\beta}H_2$), 42.8 (uC-$\underline{C}(CH_3)_2$), 46.8 (uC-CH_2NH), 54.2 (uB-$C^{\alpha}H$), 71.0 (uD-$C^{\alpha}H$), 75.8 (uA-$C^{\delta}H$), 80.1 (uA-$C^{\eta}H$), 82.3 (uA-$C^{\zeta}H$), 109.1 (uA-$\underline{C}(CH_3)_2$), 111.0 (tm, *J* = 18.6 Hz, $C_6F_5\underline{C}^{ipso}$), 124.4 (uA-$C^{\alpha}H$), 126.6, 128.6, 128.8 (uA-$C^{ar}H$), 137.4 (dm, *J* = 252.0 Hz, $C_6F_5\underline{C}^{meta}$), 137.5 (uA-$C^{ar}$), 140.2 (dm, *J* = 249.0 Hz, $C_6F_5\underline{C}^{para}$), 142.5 (uA-$C^{\beta}H$), 145.3 (dm, *J* = 242.1 Hz, $C_6F_5\underline{C}^{ortho}$), 165.5 (uA-C(O)NH), 169.3 (C(O)NH), 170.3, 177.2 (C=O); ^{19}F NMR (470 MHz, $CDCl_3$) δ −142.7 (dd, *J* = 22.6, 8.0 Hz, 2F, F^{ortho}), −155.6 (t, *J* = 20.8 Hz, 1F, F^{para}), −161.9 (td, *J* = 21.8, 7.9 Hz, 2F, F^{meta}); IR (ATR) ṽ (cm^{-1}) 2931 br w, 2359 w, 2344 w, 1752 w, 1718 w, 1676 br m, 1519 s, 1501 s, 1473 w, 1369 w, 1302 w, 1229 w, 1187 w, 1151 m, 1122 m, 1060 w, 1009 w, 973 m, 882 w, 756 m, 700 m; HRMS–MALDI $(m/z)^+$: $[M + Na]^+$ calcd for $[C_{38}H_{45}F_5N_2O_8 + Na]^+$ 775.29883; found, 775.29718.

uB-Pentafluorophenyl-cryptophycin-52 (31)

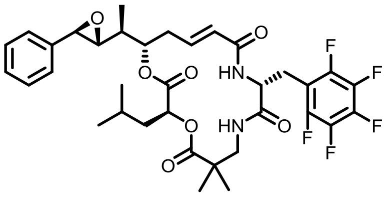

M = 694.69 g mol^{-1} $C_{35}H_{39}F_5N_2O_7$

The acetonide **30** (26 mg, 34.5 µmol) was converted into the epoxide according to GP3–6, the crude product was purified by preparative RP-HPLC (method M2), and the product was lyophilized to give 3.4 mg (4.9 µmol, 14% over 4 steps) of epoxide **31** as a voluminous, colorless solid.

HPLC: t_R 37.9 min (method M2); $[\alpha]_D^{21}$ +47.8 (*c* 0.50 in $CHCl_3$); ^{1}H NMR (600 MHz, $CDCl_3$) δ 0.85 (d, *J* = 6.0 Hz, 3H, uD-C$^{\delta}H_3$), 0.86 (d, *J* = 6.0 Hz, 3H, uD-C$^{\delta}H_3$), 1.16 (d, *J* = 6.9 Hz, 3H, uA-C$^{\varepsilon}$HC$\underline{H}_3$), 1.19 (s, 3H, uC-C($CH_3)_2$), 1.25 (s, 3H, uC-C($CH_3)_2$), 1.35 (ddd, *J* = 13.6, 8.8, 3.3 Hz, 1H, uD-C$^{\beta}\underline{H}^{A}H^{B}$), 1.69 (m, 1H, uD-C$^{\beta}H^{A}\underline{H}^{B}$), 17.3 (m, 1H, uD-C$^{\gamma}$H), 1.81 (m, 1H, uA-C$^{\varepsilon}$H), 2.47 (ddd, *J* = 14.4, 10.9, 10.9 Hz, 1H, uA-C$^{\gamma}\underline{H}^{A}H^{B}$), 2.58 (ddd, *J* = 14.4, 2.0, 2.0 Hz, 1H, uA-C$^{\gamma}H^{A}\underline{H}^{B}$), 2.94 (dd, *J* = 7.6, 2.0 Hz, 1H, uA-C$^{\zeta}$H), 2.98 (m, 1H, uB-C$^{\beta}\underline{H}^{A}H^{B}$), 3.24 (dd, *J* = 13.6, 7.1 Hz, 1H, uC-C$\underline{H}^{A}H^{B}$NH), 3.35 (dd, *J* = 13.6, 4.8 Hz, 1H, uC-C$H^{A}\underline{H}^{B}$NH), 3.45 (dd, *J* = 15.0, 4.3 Hz, 1H, uB-C$^{\beta}H^{A}\underline{H}^{B}$), 3.70 (d, *J* = 1.8 Hz, 1H, uA-C$^{\eta}$H), 4.72 (ddd, *J* = 11.0, 8.5, 4.3 Hz, 1H, uB-C$^{\alpha}$H), 4.88 (dd, *J* = 10.2, 3.4 Hz, 1H, uD-C$^{\alpha}$H), 5.18 (ddd, *J* = 11.4, 5.1, 2.0 Hz, 1H, uA-C$^{\delta}$H), 5.70 (d, *J* = 8.5 Hz, 1H, NH), 5.75 (dm, *J* = 15.1 Hz, 1H, uA-C$^{\alpha}$H), 6.75 (ddd, *J* = 15.1, 10.4, 4.7 Hz, 1H, uA-C$^{\beta}$H), 7.15 (m, 1H, NH), 7.25–7.28 (m, 2H, uA-C^{ar}H), 7.33–7.40 (m, 3H, uA-C^{ar}H); ^{13}C NMR (151 MHz, $CDCl_3$) δ 13.6 (uA-C$^{\varepsilon}$H$\underline{C}H_3$), 21.3 (uD-C$^{\delta}H_3$), 22.8 (uC-C($\underline{C}H_3)_2$), 22.9 (uC-C($\underline{C}H_3)_2$), 23.0 (uD-C$^{\delta}H_3$), 24.0 (uB-C$^{\beta}H_2$), 24.6 (uD-C$^{\gamma}$H), 37.0 (uA-C$^{\gamma}H_2$), 39.4 (uD-C$^{\beta}H_2$), 40.7 (uA-C$^{\varepsilon}$H), 42.7 (uC-$\underline{C}(CH_3)_2$), 46.8 (uC-CH_2NH), 53.4 (uB-C$^{\alpha}$H), 59.1 (uA-C$^{\eta}$H), 63.0 (uA-C$^{\zeta}$H), 71.1 (uD-C$^{\alpha}$H), 76.0 (uA-C$^{\delta}$H), 110.9 (m, $C_6F_5\underline{C}^{ipso}$), 124.7 (uA-C$^{\alpha}$H), 125.6,

128.6, 128.8 (uA-C^{ar}H), 136.7 (uA-C^{ar}), 137.5 (dm, *J* = 249.4 Hz, $C_6F_5\underline{C}^{meta}$), 140.3 (dm, *J* = 245.0 Hz, $C_6F_5\underline{C}^{para}$), 142.1 (uA-C$^{\beta}$H), 145.4 (dm, *J* = 245.4 Hz, $C_6F_5\underline{C}^{ortho}$), 165.4 (uA-C(O)NH), 169.3 (C(O)NH), 170.6, 177.4 (C=O); ^{19}F NMR (564 MHz, $CDCl_3$) δ −142.7 (dd, *J* = 22.6, 8.1 Hz, 2F, F^{ortho}), −155.3 (t, *J* = 20.9 Hz, 1F, F^{para}), −161.7 (td, *J* = 21.9, 8.0 Hz, 2F, F^{meta}); IR (ATR) ṽ (cm^{-1}) 3289 w, 2958 br w, 2177 w, 2038 w, 2010 w, 1975 w, 1748 m, 1718 m, 1662 br m, 1520 s, 1503 s, 1473 m, 1371 w, 1322 w, 1305 w, 1189 m, 1147 s, 1125 s, 1067 w, 974 m, 887 w, 752 w, 698 m; HRMS–MALDI (*m*/*z*)$^+$: [M + Na]$^+$ calcd for $[C_{35}H_{39}F_5N_2O_7 + Na]^+$ 717.25696; found, 717.25676.

4 References

1. Bodnar, B. S.; Vogt, P. F. *J. Org. Chem.* **2009,** *74,* 2598–2600. doi:10.1021/jo802778z
2. Eißler, S.; Nahrwold, M.; Neumann, B.; Stammler, H.-G.; Sewald, N. *Org. Lett.* **2007,** *9,* 817–819. doi:10.1021/ol063032l
3. Thibonnet, J.; Abarbri, M.; Parrain, J.-L.; Duchêne, A. *Tetrahedron* **2003,** *59,* 4433–4441. doi:10.1016/S0040-4020(03)00616-1
4. Bulugahapitiya, P.; Landais, Y.; Parra-Rapado, L.; Planchenault, D.; Weber, V. *J. Org. Chem.* **1997,** *62,* 1630–1641. doi:10.1021/jo961952j
5. Nahrwold, M. ß2-Aminosäuren als Bausteine funktionalisierter Cryptophycin-Analoga, Dissertation, Universität Bielefeld, 2009.
